# Engineering *Cupriavidus necator* H16 for the autotrophic production of *(R)*-1,3-butanediol

**DOI:** 10.1016/j.ymben.2021.06.010

**Published:** 2021-09

**Authors:** Joshua Luke Gascoyne, Rajesh Reddy Bommareddy, Stephan Heeb, Naglis Malys

**Affiliations:** BBSRC/EPSRC Synthetic Biology Research Centre (SBRC), School of Life Sciences, Biodiscovery Institute, The University of Nottingham, Nottingham, NG7 2RD, United Kingdom

**Keywords:** 1,3-Butanediol, 4-Hydroxy-2-butanone, Metabolic engineering, Carbon dioxide, Autotrophic fermentation, *Cupriavidus necator* H16

## Abstract

Butanediols are widely used in the synthesis of polymers, specialty chemicals and important chemical intermediates. Optically pure *R*-form of 1,3-butanediol (1,3-BDO) is required for the synthesis of several industrial compounds and as a key intermediate of β-lactam antibiotic production. The (*R*)-1,3-BDO can only be produced by application of a biocatalytic process. *Cupriavidus necator* H16 is an established production host for biosynthesis of biodegradable polymer poly-3-hydroxybutryate (PHB) *via* acetyl-CoA intermediate. Therefore, the utilisation of acetyl-CoA or its upstream precursors offers a promising strategy for engineering biosynthesis of value-added products such as (*R*)-1,3-BDO in this bacterium. Notably, *C. necator* H16 is known for its natural capacity to fix carbon dioxide (CO_2_) using hydrogen as an electron donor. Here, we report engineering of this facultative lithoautotrophic bacterium for heterotrophic and autotrophic production of (*R*)-1,3-BDO. Implementation of (*R*)-3-hydroxybutyraldehyde-CoA- and pyruvate-dependent biosynthetic pathways in combination with abolishing PHB biosynthesis and reducing flux through the tricarboxylic acid cycle enabled to engineer strain, which produced 2.97 g/L of (*R*)-1,3-BDO and achieved production rate of nearly 0.4 Cmol Cmol^−1^ h^−1^ autotrophically. This is first report of (*R*)-1,3-BDO production from CO_2_.

## Highlights

• Engineering of chemolithoautotroph *C. necator* H16 for *(R)*-1,3-butanediol production.
• Implementation of *(R)*-3-hydroxybutyraldehyde-CoA- and pyruvate-dependent pathways for *(R)*-1,3-butanediol biosynthesis.
• Redirecting carbon flux for *(R)*-1,3-butanediol biosynthesis.
• Achieved 2.97 g/L of *(R)*-1,3-butanediol with production rate of nearly 0.4 Cmol/(Cmol h) autotrophically.
• First report of *(R)*-1,3-butanediol production from carbon dioxide.

## 1 Introduction

1,3-butanediol (1,3-BDO) is an important platform chemical used in a variety of industrial applications including production of 1,3-butadiene, a precursor of synthetic rubber (Duan et al., 2016). Amongst other applications, 1,3-BDO is mainly employed in the production of unsaturated polyester resins, plasticizers, and industrial dehydrating agents. Owing to the low toxicity, and good water solubility, it can serve as a humectant and emollient in personal care products. The optically active *R*-form of 1,3-BDO is used in the production of pheromones, fragrances and insecticides (Matsuyama et al., 1993). (*R*)-1,3-BDO is also known as a precursor for most widely prescribed antimicrobial drugs, β-lactam antibiotics (Llarrull et al., 2010). Noteworthy, the 1,3-BDO can be oxidized to its ketone form 4-hydroxy-2-butanone (4H2B), an important precursor for the synthesis of pesticides, steroids, and anticancer drug doxorubicin (Zhang et al., 2010).

Chemical and biochemical synthesis methods have been developed for (*R*)-1,3-BDO production. Chemical synthesis typically yields mixture of (*R*) and (*S*) enantiomers of 1,3-BDO and requires the precursor, such as an acetaldehyde, derived from petrochemical sources (Larchevêque et al., 1991). Whereas, a more economical enzymatic biosynthesis of (*R*)-1,3-BDO has been achieved using either racemic 1,3-BDO or 4-hydroxy-2-butanone (4H2B) as substrates (Matsuyama et al., 2001). The oxido-reduction process of (4H2B) to (*R*)-1,3-BDO has been demonstrated in a variety of microorganisms such as *Kluyveromyces*, *Candida*, *Pichia*, and others, as well as engineered *Escherichia coli* (Matsuyama et al., 2001; Okabayashi et al., 2009).

With the rising concerns over carbon footprint and interest in the natural personal care products, bio-based 1,3-BDO technologies are emerging in the last decade. Microbial bioproduction of (*R*)-1,3-BDO from glucose has been first reported by Kataoka and co-workers in metabolically engineered *E. coli* (Kataoka et al., 2013). In this study, a synthetic metabolic pathway, consisting of acetyl-CoA acetyltransferase (gene *phaA*) and acetoacetyl-CoA reductase (*phaB*) from *C. necator* H16, 3-hydroxybutyryl-CoA dehydrogenase (*bld*) from *Clostridium saccharoperbutylacetonicum* N1-4(HMT) and endogenous *E. coli* NAD(P)H-dependent alcohol dehydrogenase (*yqhD*) possessing promiscuous 1,3-BDO dehydrogenase activity (Pérez et al., 2008), has been used to convert acetyl-CoA to 1,3-BDO *via* acetoacetyl-CoA, 3-hydroxybutyryl-CoA, and 3-hydroxybutanal intermediates. Optimised fed-batch fermentation using glucose as a carbon source has allowed to achieve 15.75 g/L (174.8 mmol L^−1^) of (*R*)-1,3-BDO with a 98.6% enantiomeric purity and a yield of 0.18 g/g glucose (0.37 mol/mol) (Kataoka et al., 2014). An alternative synthetic pathway has been recently investigated demonstrating conversion of pyruvate to 1,3-BDO through acetaldehyde and 3-hydroxybutanal intermediates (Kim et al., 2017; Nemr et al., 2018). Application of this pathway, consisting of pyruvate decarboxylase (PDC) from *Zimomonas mobilis*, deoxyribose-5-phosphate aldolase (Dra) from *Bacillus halodurans* and aldo/keto reductase (AKR) from *Pseudomonas aeruginosa*, has resulted in 2.4 g/L of 1,3-BDO with the yield of 56 mg/g glucose (Nemr et al., 2018).

An alternative microbial chassis that has shown great promise is chemolithoautotroph *Cupriavidus necator* H16 (formerly *Ralstonia eutropha* H16). This bacterium is able to grow aerobically and accumulate biomass to a very high level, competitive with *E. coli*, and exhibits a faster growth rate than cyanobacteria, high chemosynthetic efficiency and genetic tractability. *C. necator* H16 has been widely studied for its natural ability to produce the biodegradable polymer poly(3-hydroxybutryate) (PHB), used by this bacterium as a storage compound and accumulated in the presence of excess carbon and limited macro-elements such as nitrogen, phosphorus or oxygen (Volodina et al., 2016). *C. necator* H16 is an ideal candidate to produce platform chemicals with its ability not only to metabolise a wide range of organic compounds but more importantly to recycle CO_2_ by using the Calvin-Benson-Bassham (CBB) Cycle (Bowien and Kusian, 2002; Pohlmann et al., 2006). With the ability to fix CO_2_ as a feedstock, *C. necator* provides a significant advantage compared to the sugar-based fermentation. Besides the gasification of plant's waste, which allows the complete utilisation of carbon contained within the biomass, CO_2_, suitable for gas fermentation, can be captured from chemical plants and steel mills reducing its emission to limit the climate change (Liew et al., 2016). Considering these advantages, *C. necator* H16 has been engineered to produce a wide range of commodity chemicals including methyl ketones, alcohols, terpenes, and alka(e)nes (Bommareddy et al., 2020; Chakravarty and Brigham, 2018; Crepin et al., 2016; Grousseau et al., 2014; Krieg et al., 2018; Lu et al., 2012; Müller et al., 2013) demonstrating its versatility and potential as an industrial chassis.

**Fig. 1:**
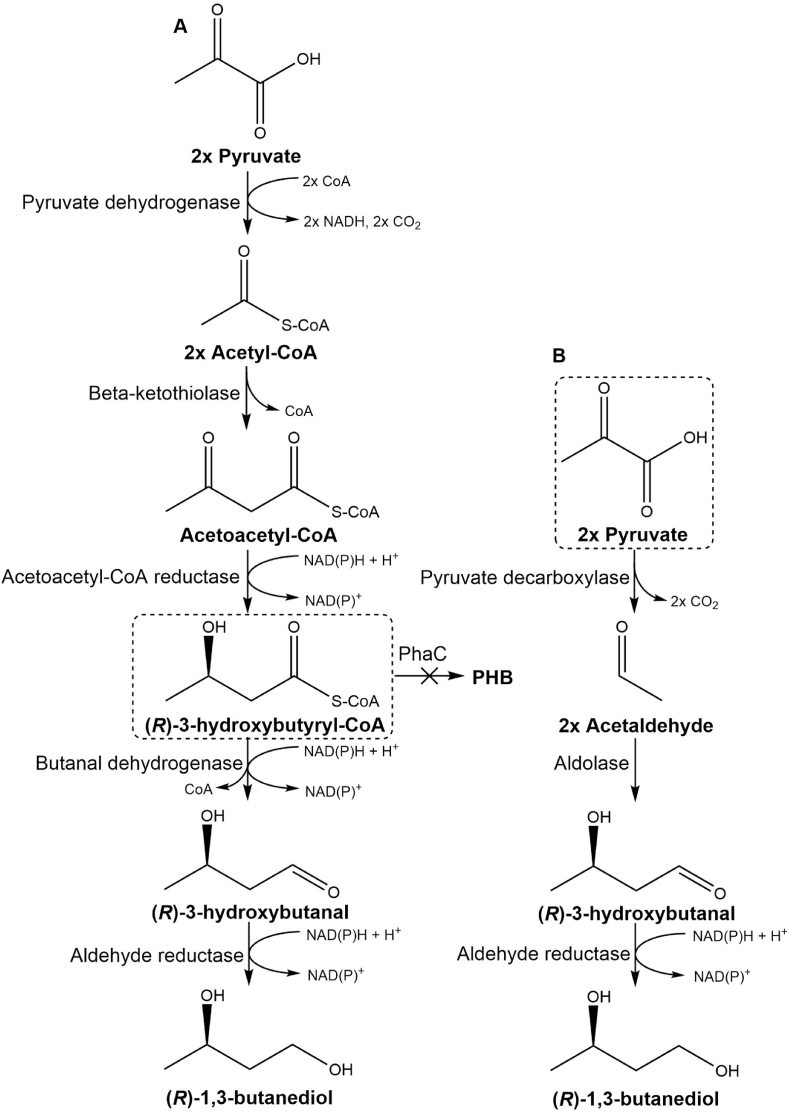
Alternative biosynthetic pathways for *(R)*-1,3-BDO production in *C. necator H16*. Required precursors 3HBCoA (A) and pyruvate (B) are highlighted with dashed line.

In this study, we aimed to engineer *C. necator* H16 for (*R*)-1,3-BDO production. Based on high availability of either (*R*)-3-hydroxybutyraldehyde-CoA ((*R*)-3HBCoA) or pyruvate precursors, two alternative (*R*)-1,3-BDO biosynthetic pathways were explored (Fig. 1). To increase (*R*)-1,3-BDO yield, a number of genetic improvements including PHB biosynthesis inactivation, redirection of the carbon flux through deletion of TCA cycle genes, and increase of the copy number of biosynthetic pathway genes were implemented. To ensure the genetic stability, both (*R*)-1,3-BDO biosynthetic pathways were chromosomally integrated in the engineered strains. Finally, autotrophic fermentation using CO_2_ as sole carbon source was demonstrated for (*R*)-1,3-BDO production.

## 2 Materials and methods

### 2.1 Gene sequences

The sequences of genes used for generation 1,3-BDO biosynthetic pathway variants were retrieved from GenBank under the following accession numbers/locus tags: AY251646 (*bld* from *C. saccharoperbutylacetonicum*); NP_417484/b3011, NP_416285/b1771, NP_417474/b3001, NP_416950/b2455, NP_415757/b1241 (*yqhD, ydjG, gpr, eutE, adhE* from *E. coli*); NP_744640/PP_2492 (*yqhD* from *Pseudomonas putida*); WP_077844196 (*s-adh* from *Clostridium beijerinckii*); CAJ92685/H16_RS07715, CAJ95981/H16_RS24705 (*gbD, hibadh* from *Cupriavidus necator*); O32210/BSU33400, P80874/BSU09530 (*yvgN, yhdN* from *Bacillus subtilius*); ADF38510/BMD_1654, ADF39485/BMD_2640, ADF40202/BMD_3362 (ADH_1_, ADH_2_, *eutE* from *Bacillus megaterium*); Q9KD67/BH1352 (*dra* from *B. halodurans*); AHJ73198/A265_01761 (PDC from *Z. mobilis*), NP_249818/PA_1127 (AKR from *P. aeruginosa*); NP_149325/CA_P0162, NP_149199/CA_P0035 (*adhE, adhE2* from *Clostridium acetobutylicum*). The *bld, adhE, dra*, *s-adh* and PDC coding sequences were optimised for *C. necator* H16 codon usage and synthesised by GeneArt Gene Synthesis (Thermo Fisher Scientific).

### 2.2 Plasmid construction

All plasmids and oligonucleotide primers used in this study are listed in Supplementary Tables 1 and 2, respectively. Plasmids were assembled using either the USER cloning method (Bitinaite et al., 2007), NEBuilder HiFi DNA assembly method (New England Biolabs) or restriction enzyme-based cloning techniques (Sambrook et al., 1989). Plasmid DNA preparation was carried out using the QIAprep® Spin Miniprep Kit (Qiagen). Gel purified linearized DNA was extracted using the QIAquick® Gel Extraction Kit (Qiagen). Genomic DNA was isolated with the GenElute™ Bacterial Kit (Sigma-Aldrich). All restriction endonucleases, T4 DNA ligase and NEBuilder® HiFi DNA Assembly Master Mix were acquired from New England Biolabs. DNA sequences were verified by Sanger sequencing (Eurofins Genomics). A detailed assembly description for each plasmid is provided in the Supplementary information.

### 2.3 Strains, transformation and media

All bacterial strains used in this study are listed in Table 1. For strain transformation, *E. coli* DH5α, MG1655 and S17-1 competent cells were prepared according to (Sambrook et al., 1989), while electrocompetent *C. necator* cells were prepared as described in (Ausubel et al., 2003).

**Table 1:** Strains used in this study. *P* denotes *P*_*araBAD*_ promoter with square brackets showing genes under promoter control.

Strain	Genotype	Parent strain	Plasmid	Source
*E. coli* MG1655	F-, λ-, *rph*-1	–	–	ATCC 70096
*E. coli* DH5α	*lacZ*ΔM15, *rec*A1, *end*A1	–	–	Invitrogen
*E.coli* S17-1	*recA pro hsdR* RP4-2-Tc::Mu-Km::Tn7	–	–	ATCC 47055
*P. putida* KT2440	wild type			ATCC 47054
*C. necator* H16	wild type	–	–	DSM-428
*C. necator* H16 phaC*^-^	PHB^−^4	–	–	DSM-541
*C. necator* H16 *ΔphaC1*	*ΔphaC1*	*-*	–	Arenas et al*.*, unpublished
*C. necator* H16 *ΔphaC1B1*	*ΔphaC1, ΔphaB1*	*-*	–	This work
*C. necator* H16 *ΔphaC1AB1*	*ΔphaC1, ΔphaA, ΔphaB1*	*-*	–	Arenas et al*.*, unpublished
*C. necator* H16 Δ2	*ΔphaC1, ΔiclAB*	–	–	This work
*C. necator* H16 Δ3	*ΔphaC1, ΔsucCD*	–	–	This work
*C. necator* H16 Δ4	*ΔphaC1, ΔiclAB, ΔsucCD*	–	–	This work
MG-p11	MG1655, (*P[bld yqhD*_*Ec*_*phaA phaB1] Km*^*r*^)	*E. coli* MG1655	pJLG11	This work
MG-p35	MG1655, (*P[bld yqhD*_*Pp*_*phaA phaB1] Km*^*r*^)	*E. coli* MG1655	pJLG35	This work
MG-p38	MG1655, (*P[bld* PA1127 *phaA phaB1] Km*^*r*^)	*E. coli* MG1655	pJLG38	This work
H16ΔC-p2	H16*ΔphaC1,* (*P[bld yqhD*_*Ec*_*] Km*^*r*^*)*	*C. necator* H16 *ΔphaC*	pJLG2	This work
H16ΔC-p15	H16*ΔphaC1,* (*P[bld yqhD*_*Pp*_*] Km*^*r*^*)*	*C. necator* H16 *ΔphaC*	pJLG15	This work
H16ΔCAB-p15	H16*ΔphaCAB, (P[bld yqhD*_*Pp*_*] Km*^*r*^*)*	*C. necator* H16 *ΔphaCAB*	pJLG15	This work
H16ΔC-p26	H16*ΔphaC1,* (*P[bld adhE2] Km*^*r*^*)*	*C. necator* H16 *ΔphaC*	pJLG26	This work
H16ΔC-p35	H16*ΔphaC1,* (*P[bld yqhD*_*Pp*_*phaA phaB1] Km*^*r*^*)*	*C. necator* H16 *ΔphaC*	pJLG35	This work
H16ΔCAB-p35	H16*ΔphaC1AB1,* (*P[bld yqhD*_*Pp*_*phaA phaB1] Km*^*r*^*)*	*C. necator* H16 *ΔphaCAB*	pJLG35	This work
H16ΔC-p304	H16*ΔphaC1,* (*P[bld yqhD*_*Ec*_*dra PDC] Km*^*r*^*)*	*C. necator* H16 *ΔphaC*	pJLG304	This work
H16ΔC-p306	H16*ΔphaC1,* (*P[ yqhD*_*Ec*_*dra PDC] Km*^*r*^*)*	*C. necator* H16 *ΔphaC*	pJLG306	This work
H16ΔCB-p14	H16*ΔphaC1,* (*P[bld] Km*^*r*^*)*	*C. necator* H16 *ΔphaC*	pJLG14	This work
H16ΔCB-p2	H16*ΔphaC1,* (*P[bld yqhD*_*Ec*_*] Km*^*r*^*)*	*C. necator* H16 *ΔphaC*	pJL2	This work
H16ΔCB-p44	H16*ΔphaC1,* (*P[bld yqhD*_*Ec*_*phaB1] Km*^*r*^*)*	*C. necator* H16 *ΔphaC*	pJL44	This work
H16Δ2-p2	H16*ΔphaC1ΔiclAB,* (*P[bld yqhD*_*Ec*_*] Km*^*r*^)	*C. necator* H16 Δ2	pJLG2	This work
H16Δ3-p2	H16*ΔphaC1ΔsucCD,* (*P[bld yqhD*_*Ec*_*] Km*^*r*^)	*C. necator* H16 Δ3	pJLG2	This work
H16Δ4-p2	H16*ΔphaC1ΔiclABΔsucCD,* (*P[bld yqhD*_*Ec*_*] Km*^*r*^)	*C. necator* H16 Δ4	pJLG2	This work
H16Δ1::54	H16*ΔphaC1::P[bld yqhD*_*Ec*_*phaAB]*	–	–	This work
H16Δ1::54-p14	H16*ΔphaC1::P[bld yqhD*_*Ec*_*phaAB],* (*P[bld] Km*^*r*^)	H16Δ1::54	pJLG14	This work
H16Δ1::54-p2	H16*ΔphaC1::P[bld yqhD*_*Ec*_*phaAB],* (*P[bld yqhD*_*Ec*_*] Km*^*r*^)	H16Δ1::54	pJLG2	This work
H16Δ1::54/Δ3::58	H16*ΔphaC1::P[bld yqhD*_*Ec*_*phaAB], ΔsucCD::P*_*8*_*[bld]*	–	–	This work
H16Δ1::56	H16*ΔphaC1::P[bld yqhD*_*Ec*_*dra PDC phaAB]*	–	–	This work
H16Δ1::56-p14	H16*ΔphaC1::P[bld yqhD*_*Ec*_*dra PDC phaAB]* (*P[bld] Km*^*r*^)	H16Δ1::56	pJLG14	This work
H16Δ1::56-p45	H16*ΔphaC1::P[bld yqhD*_*Ec*_*dra PDC phaAB]* (*P[bld dra] Km*^*r*^)	H16Δ1::56	pJLG45	This work
H16Δ1::56/Δ3::58	H16*ΔphaC1::P[bld yqhD*_*Ec*_*dra PDC phaAB], ΔsucCD::P*_*8*_*[bld]*	–	–	This work
H16Δ1::56/Δ3::60	H16*ΔphaC1::P[bld yqhD*_*Ec*_*dra PDC phaAB], ΔsucCD::P*_*8*_*[bld dra]*	–	–	This work

For heterotrophic 1,3-BDO production, *C. necator* H16 strains were grown either in minimal media (MM) containing 1 g/L NH_4_Cl, 9 g/L Na_2_HPO_4_·12H_2_O, 1.5 g/L KH_2_PO_4_, 0.2 g/L MgSO_4_·7H_2_O, 0.02 g/L CaCl_2_, 0.0012 g/L (NH_4_)_5_[Fe(C_6_H_4_O_7_)_2_] (Schlegel et al., 1961) with 1 mL/L trace element solution SL7 (25% (w/v) HCl, 0.07 g/L ZnCl_2_, 0.1 g/L MnCl_2_·4H_2_O, 0.06 g/L H_3_BO_3_, 0.2 g/L CoCl_2_·6H_2_O, 0.02 g/L CuCl_2_·2H_2_O, 0.02 g/L NiCl_2_ ·6H_2_O, 0.04 g/L Na_2_MoO_4_·2H_2_O)) (DSMZ) supplemented with 300 μg/mL kanamycin and 0.4% (w/v) sodium gluconate (C:N = 6:1); or nitrogen limiting minimal media (NLMM), which contained reduced concentration of NH_4_Cl (0.6 g/L) and 2% (w/v) (C:N = 50:1) at 30 °C and 200 rpm with orbital diameter of 1.9 cm. Overnight cultures were re-inoculated to an optical density at 600 nm (OD_600_) of 0.1 in MM or NLMM and grown for 4 h before inducing recombinant gene expression by addition of 0.01% (w/v) L(+)-arabinose, unless otherwise indicated. Initial strain screening was performed in 50-mL falcon tubes with limited aeration, whereas batch cultures for (*R*)-1,3-BDO production experiment were grown in 250-mL baffled shake-flasks with intensive aeration.

Fermentation minimal medium (FMM) was composed of following: 3.4 g/L Na_3_P_3_O_9_, 1.5 g/L NH_4_Cl, 0.5 g/L MgSO_4_, 10 mg/L CaCl_2_, 5 mg/L MnCl_2_, 50 mg/L (NH_4_)_5_[Fe(C_6_H_4_O_7_)_2_], 150 mg/L K_2_SO_4_, and 10 mL/L SL-6 trace element solution (100 mg/L ZnSO_4_, 30 mg/L MnCl_2_, 300 mg/L H_3_BO_3_, 200 mg/L CoCl_2_, 10 mg/L CuCl_2_, 20 mg/L NiCl_2_ and 30 mg/L Na_2_MoO_4_).

### 2.4 Gene knockout and knock-in generation in *C. necator*

Gene knockout and knock-in were performed using the pLO3 suicide vector exhibiting selection through tetracycline resistance (*tetR*) and counter-selection in the presence of sucrose (*sacB*). Chromosomal gene deletion was introduced by preserving start and stop codons of the gene. Where endogenous genes were replaced by introducing exogenous genes under control of the *araC*/P_*araBAD*_ inducible system, to eliminate potential transcriptional read-through, *rrnB* T2 and *rrnB* T1 terminators were incorporated upstream and downstream to the heterologous DNA region, respectively.

pLO3 suicide vector-based plasmids were transformed into *E. coli* strain S17-1 (ATCC 47055) suitable for conjugative plasmid transfer to *C. necator* H16. *E. coli* and *C. necator* strains were cultivated overnight in Luria-Bertani (LB) medium supplemented with 15 μg/mL tetracycline and 10 μg/mL gentamicin, respectively. Cells were harvested by centrifugation (5000×*g* for 10 min) and washed for mating on a LB-agar plate for 6 h at 30 °C. *C. necator* H16 transconjugants resulting from a first homologous recombination were isolated by plating onto MM-agar plates supplemented with 0.4% (w/v) sodium gluconate, 10 μg/mL gentamicin and 15 μg/mL tetracycline. Single colonies were then purified by re-streaking twice onto MM-agar plates containing gentamicin and tetracycline. Single colonies were used to inoculate 5 mL LB supplemented with gentamicin and tetracycline and cultivated overnight. Cultures were then used to inoculate 5 mL low sodium-LB (2.5 g/L NaCl) supplemented with 15% (w/v) sucrose for overnight growth. Cells were then plated onto low sodium-LB-agar plates supplemented with 15% (w/v) sucrose and single colonies were streaked onto LB-agar plates containing 15 μg/mL tetracycline and no antibiotic to establish loss of integrated chromosomal pLO3 DNA by a second homologous recombination. Cells were then screened by PCR for successful gene deletions or integrations.

### 2.5 Two-stage batch fermentation in shake-flasks

A two-stage batch fermentation in shake-flasks was employed for the production of (*R*)-1,3-BDO in *E. coli* or *C. necator*. Biomass and synthetic pathway related proteins were generated by growing cells in rich media (LB) before transferring them to nutrient limited minimal media with excess carbon. 50 μg/mL or 300 μg/mL kanamycin was used throughout for *E. coli* or *C. necator*, respectively. Freshly transformed cells from single colonies were inoculated in 5 mL of LB medium and incubated for 18 h at 30 °C and 200 rpm with orbital diameter of 1.9 cm. Subsequently, cultures of *E. coli* or *C. necator* strains were resuspended to an OD_600_ of 0.1 or 0.2 in 50 mL LB supplemented with 0.2% (w/v) glucose or 0.2% (w/v) sodium gluconate, respectively. The cultures were grown in 250 mL baffled shake-flasks at 30 °C and 200 rpm with orbital diameter of 1.9 cm. At an OD_600_ of 0.6–0.8, 0.25% (w/v) L-arabinose was added and cultures were allowed to grow further for 4–6 h enabling heterologous gene expression. Then, *E. coli* cells were harvested by centrifugation (1700*g* for 6 min), resuspended in 25 mL M9 minimal medium (0.24 mg/mL MgSO_4_, 0.011 mg/mL CaCl_2_ and M9 salts) (Sambrook et al., 1989) supplemented with 3% (w/v) glucose, 1 μg/mL thiamine and 20 μg/mL uracil (Jensen, 1993) to an OD_600_ of 10 and incubated in 250 mL baffled shake-flasks at 30 °C and 200 rpm with orbital diameter of 1.9 cm. Whereas, *C. necator* cells were harvested by centrifugation for 10 min at 6600 g, resuspended in 25 mL MM (excluding NH_4_Cl) supplemented with 2% (w/v) sodium gluconate to an OD_600_ of 7 and incubated in 250 mL baffled shake-flasks at 30 °C and 200 rpm with orbital diameter of 1.9 cm. Samples of 0.5 mL were taken immediately, 12 and 48 h after L-arabinose supplementation, centrifuged for 5 min at 17,000 g, and the cell-free supernatant was subjected to HPLC-UV/RI analysis.

### 2.6 HPLC-UV/RI analysis and chemical compound yield quantification

Prior subjecting to the HPLC-UV/RI analysis, the cell-free supernatant samples were combined with an equal volume of mobile phase (5 mM H_2_SO_4_) spiked with 50 mM valerate as internal standard, the mixture was passed through a Choice™ cellulose acetate syringe filter with 0.22 μm pore size (Thermo Fisher Scientific; cat. no. CH2213-CA) and stored in 2 mL snap cap vial closed with cap containing septa (Thames Restek; cat. no. SR-0101102-AL and SR-01011TSIT, respectively). Samples were analysed using a Thermo Scientific UltiMate 3000 HPLC system equipped with a diode array detector DAD-3000 with the wavelengths set at 210 nm and 280 nm, a refractive index detector RefractoMax 521 (Thermo Fisher Scientific), and Phenomenex Rezex ROA-organic acid H+ (8%) 150 mm × 7.8 mm × 8 μm column (Phenomenex). The column was operated at 35 °C with an isocratic flow rate of 0.5 mL/min. Samples were run for 30 min and the injection volume was 20 μl. Chromeleon Chromatography Data System software was used for HPLC system control, data processing and analysis. The concentrations of chemical compounds were estimated from standard calibration curves generated by analysing known concentrations of sodium gluconate (cat. no. 10356290) and ethanol (cat. no. 10437341) from Fisher Scientific; 4-hydroxy-2-butanone (Alfa Aesar; cat. no. L11456); 3-hydroxybutyraldehyde (Aldol; cat. no. CDS019977) and acetic acid (cat. no. A6283) from Sigma-Aldrich; L-arabinose (cat. no. 365185000), 1,3-butanediol (99% purity, Cat. No. 107622500) and pyruvic acid (cat. no. 132145000) from Arcos Organics.

Chemical compound yields per biomass (Y_P/X_) and substrate (Y_P/S_) were calculated using equations (1), (2), respectively:

(1) $$Y_{P/X}=\frac{P_{t}^{*}-P_{t-1}^{*}}{\left(X_{t}+X_{t-1}\right)/2}$$

where P*_t_ and P*_t-1_ are concentrations of chemical compound (e.g. 1,3-BDO) in g/L for time points *t* and *t-1*, *X*_*t*_ and *X*_*t-1*_ are dry cell weight concentrations in g/L for time points *t* and *t-1*.

(2) $$Y_{P/S}=\frac{P_{t}-P_{t-1}}{S_{t}-S_{t-1}}$$

where *P*_*t*_ and *P*_*t-1*_ are concentrations of chemical compound in carbon mole (Cmol) for time points *t* and *t-1*, *S*_*t*_ and *S*_*t-1*_ are concentrations for substrate sodium gluconate in Cmol for time points *t* and *t-1*.

To estimate dry cell weight (DCW), 1 mL of cell culture was centrifuged in pre-dried and pre-weighed 1.5 mL Eppendorf tubes for 2 min at 17000 g and the supernatant was discarded. The cell pellet was dried for 48 h at 120 °C in a Heratherm OGH60 gravity convection oven (Thermo Fisher Scientific). Subsequently, samples were cooled in a desiccator and the DCW was determined using an analytical balance with accuracy to 0.1 mg (SI-234, Denver Instrument). DCW was calculated as grams per litre.

### 2.7 Specific cell growth rate

Cell growth was monitored by measuring the OD_600_ using a BioMate™ 3S UV–Visible Spectrophotometer (Thermo Fisher Scientific, MA, USA). Specific growth rate (μ) was calculated using the following equation (Widdel, 2007).

(3) $$\mu \left(t\right)=\frac{\text{ln}OD_{1}-\text{ln}OD_{0}}{\left(t_{1}-t_{0}\right)}$$

where ln*OD*_1_ and ln*OD*_0_ are the calculated natural logarithm values of measured OD_600_ for time points *t*_1_ and *t*_0_.

### 2.8 Fermentation

Autotrophic fermentation was carried out in 1.3 L vessel using a DASGIP® parallel bioreactor 4-fold system with Bioblock for microbiology including control modules CWD4, MP8, PH4PO4L, PH4PO4RD4, OD4, MX4/4, TC4SC4 (Eppendorf) equipped with probes to measure dissolved oxygen (DO) (optical DO probe, Mettler Toledo), pH (405-DPAS-SC-K8S pH Probe, Mettler Toledo) and temperature Platinum RTD Temperature Sensor (Eppendorf). DASware® control software was used for automated control of DO, temperature, and pH. The preculture was prepared and fermentation was performed as described previously (Bommareddy et al., 2020) with some modifications. Briefly, The first seed culture was grown overnight at 30 °C with 200 rpm shaking in 10 mL of LB from a single colony. Subsequently, this culture was reseeded to 120 mL of LB and grown for another 24 h as above. Resulting cells were harvested by centrifugation for 10 min at 6600*g*, washed with 10 mL of FMM to remove residual LB, resuspended in 50 mL FMM and used to inoculate 700 mL FMM. If appropriate, antibiotics were added to the growth medium at the following concentrations: 10 μg/mL gentamicin or 300 μg/mL kanamycin. When cells reached DCW greater than 1 g/L protein expression was induced by addition of L-arabinose. pH was controlled at 6.9 by the addition of 1 M NH_3_OH until a DCW of 0.75 g/L was achieved, changing to 1 M KOH to limit nitrogen availability. DO was maintained at 10% (v/v) by increasing air flow (8.5–9.5 L/h) and agitation with a Rushton-type impeller (400–1600 rpm) and temperature at 30 °C. Using the DASGIP MX 4/4 Gas Mixing Module CO_2_, H_2_ and air were continuously sparged through 0.22 μm membrane filters into the bioreactors. Gas outflow composition was analysed using a Bioprocess R&D Lab Gas Analyser, Model RLGA-9804 (Atmosphere Recovery Inc.). 2 mL samples were taken immediately after addition of L-arabinose and then every 12 h for 120 h and subjected to the HPLC-UV/RI analysis.

## 3 Results and discussion

### 3.1 Choice of (*R*)-1,3-BDO biosynthetic pathways

The systematic approach to engineer *C. necator* H16 for 1,3-BDO production was based on the following design and experimental rationale: 1) considering alternative biosynthetic pathways which enable to utilise pyruvate and its downstream anabolic products as precursors; 2) screening enzymes with butanal dehydrogenase and aldehyde reductase activities enabling biosynthesis of 1,3-BDO from (R)-3-hydroxybutyraldehyde-CoA, the natural pyruvate's anabolic product in *C. necator*; 3) engineering *C. necator* H16 strain to improve the flux towards precursors required for 1,3-BDO biosynthesis; 4) establishing fermentation conditions and strain engineering to reduce the by-product biosynthesis; 5) ultimately, developing *C. necator* H16 strain suitable for production 1,3-BDO from CO_2_.

*C. necator* H16 lacks any phosphofructokinase (2.7.1.11; 2.7.1.90 or 2.70.1.146) of the Embden-Meyerhoff-Parnas (EMP) pathway and 6-phosphogluconate dehydrogenase (1.1.1.44 or 1.1.1.343) of the oxidative pentose phosphate (OPP) pathway. Such organisation of metabolism restricts the flux through OPP and forward-EMP pathways and instead directs it through the Entner–Doudoroff pathway under heterotrophic growth conditions. Under autotrophic conditions, CO_2_ is fixed by the reductive pentose phosphate cycle into the glyceraldehyde-3-phosphate and can increase the carbon flux through the reversed-EMP and ED pathways, as this has been observed under mixotrophic growth conditions (Alagesan et al., 2018b). The resultant flux distribution increases the availability of pyruvate that is used as a precursor for PHB synthesis in *C. necator* H16 under excess carbon and limited macro-elements conditions (Volodina et al., 2016).

Consequently, based on this existing knowledge, the pyruvate was identified as a highly available precursor for 1,3-BDO biosynthesis in *C. necator* H16. Two alternative heterologous biosynthetic pathways that branches out from pyruvate were considered: A) utilising (*R*)-3-hydroxybutyraldehyde-CoA ((*R*)-3HBCoA) and requiring two heterologous enzymatic reactions: (i) deacylation of (*R*)-3HBCoA to (*R*)-3-hydroxybutanal ((*R*)-3HBA) by butanal dehydrogenase (CoA-acylating, NADH-dependent) (Bld, EC 1.2.1.57), and (ii) reduction of (*R*)-3HBA into (*R*)-1,3-BDO by NADPH-dependent aldehyde reductase activity (YqhD, EC 1.1.1.2) (Pérez et al., 2008); B) utilising pyruvate and requiring three heterologous enzymatic reactions: (i) decarboxylation of pyruvate to acetaldehyde by pyruvate decarboxylase (Pdc, EC 4.1.1.1), (ii) condensation of two acetaldehyde molecules to (*R*)-3HBA by deoxyribose-5-phosphate aldolase (Dra/DeoC, EC 4.1.2.4); and (iii) reduction of (*R*)-3HBA into (*R*)-1,3-BDO by NADPH-dependent aldehyde reductase (Fig. 1). Evidently, the same enzymatic activity can be utilised for the final conversion of (*R*)-3HBA to (*R*)-1,3-BDO in both pathways.

The (*R*)-3HBCoA pathway requires three NAD(P)H, whereas the pyruvate pathway utilises one NADPH with two NAD^+^ molecules remaining in oxidized form due to the direct conversion of pyruvate into acetaldehyde. Both pathways are NAD(P)H-consuming with net use of three reducing cofactor molecules for each (*R*)-1,3-BDO synthesised, and are, therefore, heavily reliant on the efficient regeneration and balance of reducing equivalent within the cell. Indeed, Bld protein contains a proline and a nonpolar/aliphatic amino acid in sequence positions that correspond to the residues P222 and I257 of structurally similar PduP (Supplementary Figure 1), which are implicated in the selectivity for NADH over NADPH (Trudeau et al., 2018). Moreover, *in vitro* assays have shown that Bld possess the NADH-dependent activity (Hwang et al., 2014).

Previous research has shown that key TCA cycle genes (*sucC, fumA, mdh1*) are downregulated when *C. necator* cells transition from exponential to stationary growth phase alongside the upregulation of PHB required genes *phaAB* (Peplinski et al., 2010): as one of a key nutrient is depleted and biomass production becomes restricted, the flux through *(R)*-3HBCoA is increased and the carbon is accumulated in the form of PHB. This involves β-ketothiolase (PhaA), NADP-dependent acetoacetyl-CoA reductase (PhaB) and poly(3-hydroxyalkanoate) polymerase (PhaC) activities. Notably, the PHB can constitute up to 90% of the DCW, if the excess carbon is available under nitrogen-limiting conditions (Volodina et al., 2016). This strongly suggests that a sufficiently large pool of precursor in form of 3HBCoA can be generated under nutrient-limiting conditions generating a driving force for (*R*)-1,3-BDO biosynthesis when the (*R*)-3HBCoA-dependent pathway is utilised. Moreover, the deletion of *phaC1* gene significantly reduces the poly(3-hydroxyalkanoate) polymerase activity enabling accumulation of *(R)*-3HBCoA, which can be utilised for biosynthesis of (*R*)-1,3-BDO.

Therefore, the (*R*)-3HBCoA-dependent (*R*)-1,3-BDO biosynthetic pathway was primarily selected for (*R*)-1,3-BDO production in *C. necator H16* heterotrophically or from CO_2_. The PHB deficient Δ*phaC1* strain was utilised for the (*R*)-3HBCoA-dependent pathway implementation and further metabolic engineering.

### 3.2 Implementation of (*R*)-3HBCoA-dependent (*R*)-1,3-BDO biosynthetic pathway

#### 3.2.1 Screening of biosynthetic pathway variants

To enable implementation of (*R*)-3HBCoA-dependent (*R*)-1,3-BDO biosynthetic pathway, a screening of gene combinations, encoding enzymes with butanal dehydrogenase and aldehyde reductase activities, was performed (Supplementary Figure 2).

In this screen, as a substitute for the bifunctional AdhE2 from *C. acetobutylicum* (Fontaine et al., 2002), a butanal dehydrogenase (Bld) from *C. saccharoperbutylacetonicum* (Kosaka et al., 2007; Nair et al., 1994) was combined with a number of aldehyde reductases, including widely utilised YqhD from *E. coli* (Jarboe, 2011). The *bld* gene possessing a very low GC content of 32.8% was codon-optimised for expression in *C. necator* H16 (66.3% average GC content). Aldehyde reductase candidates were selected based on protein homology to YqhD or enzymatic activity on similar compounds reported previously, such as the conversion of 4-hydroxybutyraldehyde to 1,4-butanediol (Wang et al., 2017), acetoin to 2,3-butanediol (Yan et al., 2009) or the *in vitro* conversion of 3-hydroxybutyraldehyde to 1,3-butanediol (Kim et al., 2017). Furthermore, the *yqhD* gene was combined with *eutE* from two different species, as well as *adhE2* and *adhE1* from *C. acetobutylicum* and *E. coli adhE* were included.

All pathway variants were tested in *C. necator* H16 wild-type and PHB deficient mutant with the (*R*)-1,3-BDO biosynthesis observed only in the latter. (*R*)-1,3-BDO was produced in strains H16ΔC-p2, H16ΔC-p15 and H16ΔC-p26 expressing *bld* with *yqhD* from *E. coli* MG1655 (hereafter denoted as *yqhD*_*Ec*_) or *P. putida* KT2440 (*yqhD*_*Pp*_), and bifunctional *adhE2* from *C. acetobutylicum*, respectively (Supplementary Figure 2). Other biosynthetic pathway variants did not show detectable quantities of the diol by HPLC-RI*.* Biosynthesis of (*R*)-1,3-BDO in *C. necator* H16 obtained using bifunctional *adhE2* on its own or *bld* in combination with *yqhD* is consistent with previously reported activities of these enzymes (Hwang et al., 2014; Kataoka et al., 2013) confirming their indispensable role.

It should be noted that (*R*)-1,3-BDO exhibited only a minor toxic effect on *C. necator* H16 with no growth inhibition in the presence of up to 83.2 mM (Supplementary Table 3).

#### 3.2.2 Evaluation of (*R*)-1,3-BDO biosynthesis

Previous research has shown that the PHB synthesis in *C. necator* H16 is increased under nitrogen limiting conditions with excess carbon available (Tian et al., 2005). The nitrogen limitation effect on (*R*)-1,3-BDO yield was investigated H16ΔC-p26 by changing carbon/nitrogen (C/N) ratio in culture minimal medium from 6 to 50. In spite of decrease in growth rate, more than 2-fold higher yield of (*R*)-1,3-BDO was observed using C/N ratio of 50 (Supplementary Figure 3). Furthermore, Y_1,3-BDO/S_ of 0.018 was measured 24 h after induction under nitrogen limitation, whereas in non-limiting nitrogen conditions (*R*)-1,3-BDO became detectable only after 48 h. These results demonstrate that (*R*)-1,3-BDO biosynthesis in *C. necator* H16 can be improved by limiting nitrogen availability.

The AraC/P_*araBAD*_*-*arabinose inducible system is relatively well repressed under uninduced state and can be fine-tuned in the range from 0.00117 to 0.15% (w/v) of L-arabinose allowing to achieve more than 1000-fold induction in *C. necator* H16 (Alagesan et al., 2018a). Importantly, the L-arabinose is not metabolised by this bacterium and does not exhibit any adverse effect on the cell growth (data not shown). Therefore, AraC/P_*araBAD*_ inducible system was selected to drive overexpression of (*R*)-1,3-BDO biosynthesis genes.

To establish an optimal gene expression level of (*R*)-3HBCoA-dependent (*R*)-1,3-BDO biosynthetic pathway, induction conditions using a range of L-arabinose concentrations (from 0.005 to 0.2% (w/v)) were investigated. For H16ΔC-p2 strain expressing *bld* and *yqhD*_*Ec*_, a direct correlation between the biosynthesis levels of (*R*)-1,3-BDO and concentration of inducer was observed 24 h after induction with the highest quantity of (*R*)-1,3-BDO produced in the cell culture that was supplemented with 0.2% of L-arabinose (Supplementary Figure 4). However, at the later stages of induction, the specific (*R*)-1,3-BDO production was reduced and a strong growth inhibition observed in cultures supplemented with higher than 0.045% concentrations of L-arabinose. Altogether these results revealed that 0.01–0.045% concentrations of L-arabinose are optimal for induction of (*R*)-1,3-BDO biosynthetic pathway genes when the plasmid-based expression system is used in *C. necator* H16.

**Fig. 2:**
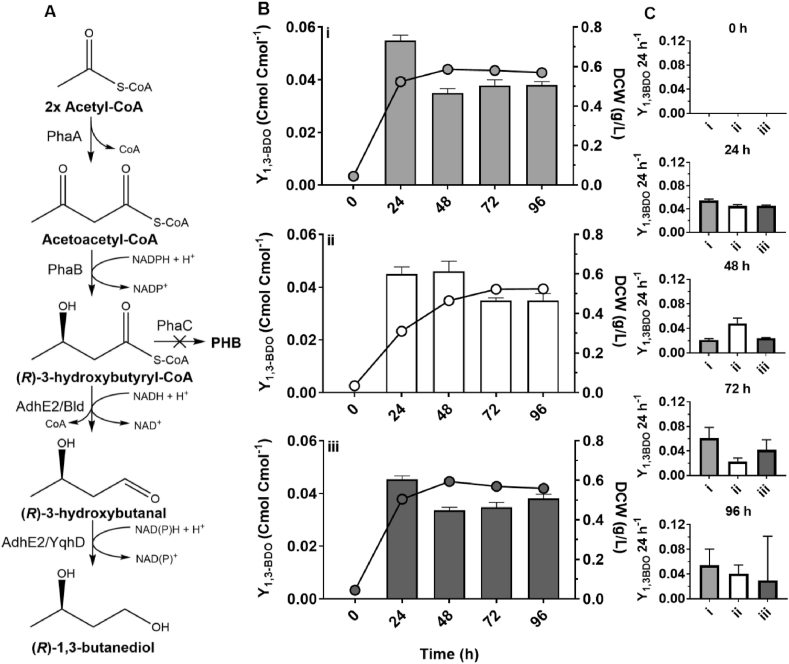
Comparison of (*R*)-1,3-BDO yields in *C. necator* expressing alternative genes of *(R)-*3HBCoA-dependent pathway. (A) The endogenous β-ketothiolase (PhaA) and NADP-dependent acetoacetyl-CoA reductase (PhaB) provides (R)-3HBCoA, a precursor metabolite, which is converted by AdhE2 or a combination of Bld and YqhD into (*R*)-1,3-BDO. The *phaC1* encoding a poly(3-hydroxyalkanoate) polymerase (PhaC) for PHB synthesis is chromosomally knocked-out to re-direct metabolic flux towards (*R*)-1,3-BDO. (B) Carbon yield of (*R*)-1,3-BDO (bars) and dry cell weight (circles) in *C. necator* strains H16ΔC-p2 (i), H16ΔC-p15 (ii) and H16ΔC-p26 (iii) 0, 24, 48, 72 and 96 h after the induction of heterologous gene expression with 0.01% (w/v) L-arabinose. Yields calculated from time-point 0 (C) Carbon yield of (*R*)-1,3-BDO within specific 24-h time periods. Yields calculated from the previous time-point. Cells were grown in 2% (w/v) sodium gluconate NLMM using 250 mL baffled shake flasks. Results represent the average of three biological replicates and error bars show standard deviation.

Next, (*R*)-1,3-BDO-producing *C. necator* strains H16ΔC-p2, H16ΔC-p15 and H16ΔC-p26 were compared under heterotrophic nitrogen-limited growth conditions (Fig. 2). Cumulative yields of (*R*)-1,3-BDO were steady for the duration of 96-h cell growth period ranging from 0.035 to 0.055 Cmol Cmol^−1^, and comparable between all three strains. Increase in biomass and 1,3-BDO was greatest during initial 24-h post induction period with a highest yield of 0.055 ± 0.003 Cmol Cmol^−1^ obtained using strain H16ΔC-p2. It can be concluded that of three strains possessing alternative combinations of genes of *(R)-*3HBCoA-dependent pathway, the strain H16ΔC-p2 performed marginally better producing highest yields of (*R*)-1,3-BDO during early logarithmic and stationary growth periods while exhibiting the least growth impairment. Furthermore, YqhD_*Ec*_ aldehyde reductase specificity on butanal is higher (Km = 0.67) than that of AdhE2 (Km = 1.60) as reported previously (Palosaari and Rogers, 1988; Pérez et al., 2008). Therefore, the combination of genes *bld* and *yqhD*_*Ec*_ was chosen to be utilised for *(R)-*3HBCoA-dependent biosynthetic pathway in next stages of this study.

#### 3.2.3 YqhD facilitates higher *(R)*-1,3-BDO yield in *C. necator*

A NADPH-dependent aldo-keto reductase (AKR, *PA1127*) from *P. aeruginosa* has been shown to convert 3-hydroxybutanal into (*R*)-1,3-BDO (Kim et al., 2017) enabling to achieve yield of 0.075 Cmol Cmol^−1^-glucose in *E. coli* (Nemr et al., 2018). To compare the efficiency of AKR for (*R*)-1,3-BDO production in *E. coli* MG1655 and *C. necator* H16, plasmid constructs containing *PA1127* replacing *yqhD* were assembled. Then, the *(R)*-1,3-BDO biosynthesis was achieved using two-stage batch fermentation in the 250 mL baffled shake flask as described in *Materials and Methods*. *E. coli* cells harbouring plasmid pJLG38 (MG-p38 containing *PA1127*) or pJLG11 (MG-p11 containing *yqhD*_*Ec*_) and *C. necator* strains harbouring plasmids with either *PA1127* (H16ΔC-p20) or *yqhD*_*Ec*_ (H16ΔC-p2) were cultivated in rich media and heterologous gene expression was induced by supplementing media with 0.25% (w/v) of L-arabinose, allowing biomass and recombinant enzyme production. Cells were then resuspended to a high cell density in minimal media with an abundance of either glucose (*E. coli*) or sodium gluconate (*C. necator*), cultured for 48 h and (*R*)-1,3-BDO concentration was measured in the media. *E. coli* MG-p38 strain harbouring plasmid with *PA1127* gene yielded 0.087 (*R*)-1,3-BDO (Cmol Cmol^−1^) (Table 2) supporting previous work (Nemr et al., 2018). Whereas, *C. necator* strain H16ΔC-p20 with *PA1127,* produced almost 2-fold less of (*R*)-1,3-BDO. Strikingly, *C. necator* strain H16ΔC-p2 expressing *yqhD*_*Ec*_ achieved the highest (*R*)-1,3-BDO yield of 0.140 (Cmol Cmol^−1^). Notably, similar improvement in the production of diols and other reduced chemical compounds using two-stage fermentation approach has been reported previously (Burg et al., 2016; Kataoka et al., 2013; Nemr et al., 2018).

**Table 2:** Concentration of (*R*)-1,3-BDO and yields of (*R*)-1,3-BDO and 4H2B obtained using two-stage batch fermentation by *E. coli* MG-p11 and MG-p38 or *C. necator* H16ΔC-p2 and H16ΔC-p20 strains. Values represent the average and standard deviation of three biological replicates.

Strain	(*R*)-1,3-BDO (mM)	Y_1,3BDO_ (Cmol Cmol^−1^)	Y_4H2B_ (Cmol Cmol^−1^)
*E. coli* MG-p11	1.994 ± 0.504	0.035 ± 0.010	N.D.
*E. coli* MG-p38	8.903 ± 0.239	0.087 ± 0.019	N.D.
*C. necator* H16ΔC-p2	14.805 ± 0.454	0.140 ± 0.002	0.030 ± 0.001
*C. necator* H16ΔC-p20	5.311 ± 0.289	0.048 ± 0.003	0.024 ± 0.001

Overexpression of *yqhD* has a clear adverse effect on the *(R)-*1,3-BDO yield in *E. coli* but not in *C. necator*. This is likely due to acetaldehyde dehydrogenase activity causing production of ethanol as reported previously (Nemr et al., 2018) and with this associated depletion of NADPH.

#### 3.2.4 Metabolic by-products of the *(R)*-3HBCoA-dependent pathway

As indicated in section 3.1, *C. necator* H16 strains with Δ*phaC1* background were primarily used for biosynthesis of (*R*)-1,3-BDO. Further analysis of extracellular metabolite composition revealed that, alongside the *(R)*-1,3-BDO, large amounts of pyruvate, representing yields of 0.419 ± 0.003 Cmol Cmol^−1^, 0.505 ± 0.008 Cmol Cmol^−1^ and 0.415 ± 0.011 Cmol Cmol^−1^, were respectively excreted from strains H16ΔC-p2, H16ΔC-p15 and H16ΔC-p26, containing *(R)*-3HBCoA-dependent pathway variants. Whereas only negligible quantities of acetate and ethanol were detected in these strains. The pyruvate was completely absent in cultures of wild-type background strains harbouring same biosynthetic pathway variants. The accumulation and excretion of pyruvate has been reported previously in *C. necator* H16 *ΔpdhL* and PHB^−^4 (DSM541) strains (Raberg et al., 2011; Steinbüchel and Schlegel, 1989). The former is deficient of the dihydrolipoamide dehydrogenase (E3) component of pyruvate dehydrogenase complex. The accumulation of pyruvate in PHB^−^ strains indicates that the deficiency of poly(3-hydroxyalkanoate) polymerase activity causes the build-up of upstream metabolites of the PHB pathway and that the increase in acetyl-CoA level inhibits the pyruvate dehydrogenase activity, as postulated previously (Jung and Lee, 1997; Raberg et al., 2011; Steinbüchel and Schlegel, 1989). Simultaneously, the pyruvate accumulation suggests that the *(R)*-3HBCoA-dependent pathway exhibits limited capacity to drive carbon flux towards the *(R)*-1,3-BDO.

**Fig. 3:**
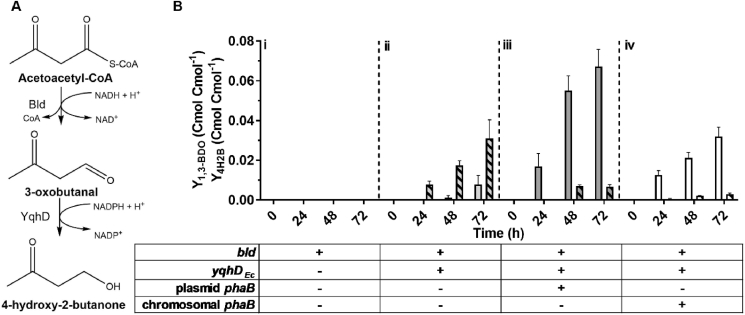
Biosynthesis of 4H2B in *C. necator* expressing heterologous *bld* and *yqhD* genes. (A) Schematic of the 4H2B biosynthetic pathway. Acetoacetyl-CoA is converted to 3-oxobutanal by Bld exhibiting promiscuous acylating dehydrogenase properties. Then, 3-oxobutanal is subsequently reduced to 4H2B by YqhD. (B). 1,3-BDO carbon yield (bars) and 4H2B carbon yield (striped bars) in batch fermentation cultures of H16ΔCB-p14 (i), H16ΔCB-p2 (ii), H16ΔCB-p44 (iii) and H16ΔC-p2 (iv). Plus or minus sign indicates the presence or absence of a gene. Results represent the average of three biological replicates and error bars show standard deviation.

Alongside with the *(R)*-1,3-BDO synthesis and accumulation of pyruvate, the 4-hydroxy-2-butanone (4H2B) was observed as a by-product in engineered *C. necator* H16 expressing *(R)*-3HBCoA-dependent biosynthetic pathway genes. As shown previously, the butanal dehydrogenase Bld exhibits enzymatic activity on various C4-CoA derivatives including 3HBCoA and 4HBCoA (Hwang et al., 2014; Kataoka et al., 2013). Therefore, we hypothesized that this promiscuous enzyme can also act upon the excess acetoacetyl-CoA (3-oxobutyryl-CoA), produced by the β-ketothiolase, PhaA, converting it into 3-oxobutanal, which is further transformed into 4H2B by YqhD promiscuous activity (Fig. 3A). To test this hypothesis, 4H2B and *(R)*-1,3-BDO biosynthesis was evaluated in *C. necator* Δ*phaC1B1* strain transformed either with plasmid pJLG14 containing *bld* (strain H16ΔCB-p14); pJLG2 with *bld* and *yqhD*_*Ec*_ (H16ΔCB-p2) or pJLG44 containing *bld*, *yqhD*_*Ec*_ and *phaB* genes (H16ΔCB-p44). Results in Fig. 3B show that neither *(R)*-1,3-BDO nor 4H2B are detectable in the culture of H16ΔCB-p14 when *yqhD* activity is absent. However, both compounds are synthesised by H16ΔCB-p2 and H16ΔCB-p44 containing both *bld* and *yqhD* genes. Moreover, in the absence of *phaB1* gene (strain H16ΔCB-p2), mostly 4H2B is synthesised, whereas strains H16ΔCB-p44 and H16ΔC-p2, possessing *bld*, *yqhD*_*Ec*_ and *phaB1* genes, produce predominantly *(R)*-1,3-BDO (Fig. 3B). These results confirm that when NADP-dependent acetoacetyl-CoA reductase activity is reduced by deletion of *phaB1* gene, the acetoacetyl-CoA accumulates and is subsequently converted into 4H2B by Bld and YqhD activities. Notably, even if *phaB1* gene is absent, a small quantity of *(R)*-1,3-BDO is generated, most likely through the activity of other *C. necator* PhaB homologues encoded by *phaB2* and *phaB3*. When Δ*phaB1* is complemented with plasmid-based *phaB* (H16ΔCB-p44), the *(R)*-1,3-BDO biosynthesis is recovered, whereas the 4H2B yield is drastically reduced, indicating increase in availability of *(R)*-3HBCoA for conversion into the diol by Bld and YqhD. Overall, these results suggest that by-product's 4H2B formation can be reduced by improving expression or copy number of *phaB1* encoding for NADP-dependent acetoacetyl-CoA reductase. On the another hand, the 4H2B can be converted into *(R)*-1,3-BDO using enzymes with reducing activity as identified previously (Matsuyama et al., 2001; Okabayashi et al., 2009).

### 3.3 Improvement of *(R)*-1,3-BDO production in *C. necator*

#### 3.3.1 Overexpression of endogenous *phaA* and *phaB1*

**Fig. 4:**
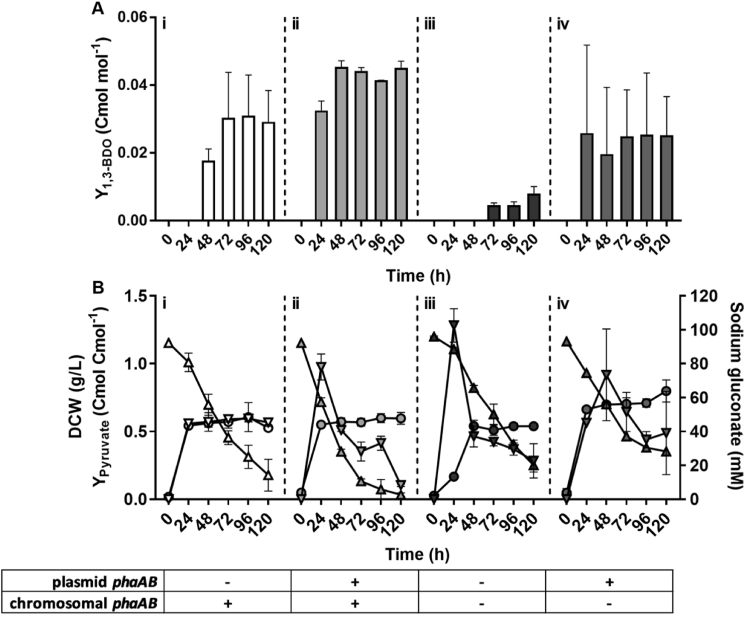
Improvement of *(R)*-1,3-BDO production by overexpression of *phaAB*. Batch fermentation profile data for strains H16ΔC-p15 (i); H16ΔC-p35 (ii); H16ΔCAB-p15 (iii) and H16ΔCAB-p35 (iv) are presented as following: (A) *(R)*-1,3-BDO yield (bars), (B) biomass DCW (circles) sodium gluconate concentration (triangles) and pyruvate yield (upside down triangles). Cells were grown in NLMM supplemented with 2% (w/v) sodium gluconate. The gene expression was induced by addition of 0.01% (w/v) arabinose. Results represent the average of three biological replicates and error bars show standard deviation.

The endogenous *C. necator* H16 genes *phaA* and *phaB* are essential for biosynthesis of 3-HBCoA from acetyl-CoA (Fig. 1). In order to assess whether enhanced expression of *phaA* and *phaB* by increasing their copy number and expression level can improve *(R)*-1,3-BDO production, *phaA* and *phaB* in addition to *bld* and *yqhD* genes were included in the plasmid-based overexpression system yielding pJLG35. The yield of *(R)*-1,3-BDO in H16ΔC-p35 containing chromosomal and plasmid-based copies of *phaAB* was compared to that in H16ΔC-p15 (chromosomal copy of *phaAB*), H16ΔCAB-p15 (no *phaAB*) and H16ΔCAB-p35 (plasmid-based only copy of *phaAB*) (Fig. 4). Of all strains, H16ΔC-p35 and H16ΔCAB-p35 exhibited the diol production within the first 24 h, whereas the former maintained highest yield (approximately 0.045 Cmol Cmol^−1^) throughout the rest of 120-h fermentation. Evidently, expression of plasmid-based *phaAB* genes encoding acetoacetyl-CoA reductase improved utilisation of carbon source and conversion of pyruvate at the later stages of fermentation. Interestingly, despite the lack of *phaAB* in strain H16ΔCAB-p15, the production of *(R)*-1,3-BDO was still observed, albeit at much lower yields of 0.008 ± 0.003 Cmol Cmol^−1^. Specific production of 1,3-BDO by strain H16ΔCAB-p15 after 120 h indicates combined activity of one or multiple β-ketothiolase homologues reported in the *C. necator* genome (Lindenkamp et al., 2012) and acetoacetyl-CoA reductase PhaB3 (H16_A2171) possessing reduced rate compared to PhaB1 (Budde et al., 2010).

#### 3.3.2 Reducing TCA cycle flux for enhanced 1,3-BDO production

**Fig. 5:**
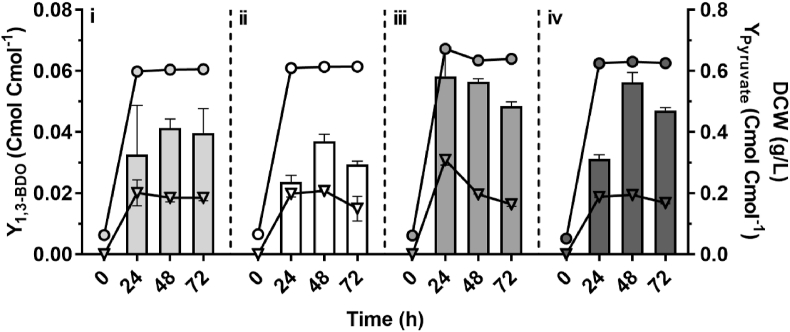
Improvement of *(R)*-1,3-BDO yields by *sucCD* deletion. Yields of *(R)*-1,3-BDO (bars) and pyruvate (upside down triangles), and DCW obtained using strains H16ΔC-p2 (i), H16Δ2-p2 (ii), H16Δ3-p2 (iii), H16Δ4-p2 (iv) are shown. Cells were grown in NLMM supplemented with 2% (w/v) sodium gluconate. The biosynthetic pathway gene expression was induced by addition of 0.01% (w/v) arabinose. Results represent the average of at least two biological replicates and error bars show standard deviation.

With previous literature detailing improvement of PHB production as a result of acetyl-CoA accumulation facilitated through gene deletions observed in *E. coli* (Centeno-Leija et al., 2014), *C. necator* genes *sucCD* and *iclAB* were targeted to be deleted individually and in combination to reduce TCA cycle carbon flux, increasing acetyl-CoA pool for 1,3-BDO production (Fig. 5). *C. necator* strains H16 *ΔphaCΔiclAB* (H16Δ2), H16 *ΔphaCΔsucCD* (H16Δ3) and H16 *ΔphaCΔiclABΔsucCD* (H16Δ4) were generated. For *(R)*-1,3-BDO yield profiling, they were transformed with plasmid pJLG2 containing (*R*)-3HBCoA-dependent (*R*)-1,3-BDO biosynthetic pathway genes, batch fermentation and product analysis performed as above. The results showed that the overall (*R*)-1,3-BDO yield was significantly higher for engineered strains H16Δ3-p2 and H16Δ4-p2. Notably, H16Δ3-p2 exhibited nearly 2-fold higher yield than other strains after 24 h of fermentation.

As predicted, deletion of *sucCD* helped to improve (*R*)-1,3-BDO yield likely through increased acetyl-CoA pool. Despite the loss of ATP generation by the deletion of *sucCD*, all strains exhibited similar specific growth rates. Indistinctly, *iclAB* deletion strains showed no improvement in (*R*)-1,3-BDO biosynthesis as the sole deletion or when combined with *sucCD* deletion. Since *iclAB* has been previously reported to be primarily involved in β-oxidation pathways (Brigham et al., 2010; Sharma et al., 2016) and only low expression level was observed under heterotrophic growth (Alagesan et al., 2018b) this indicates its reduced involvement in gluconate metabolism and flux through the glyoxylate bypass.

### 3.4 Implementation of pyruvate-dependent *(R)*-1,3-BDO pathway

#### 3.4.1 Evaluation of pyruvate-dependent pathway

**Fig. 6:**
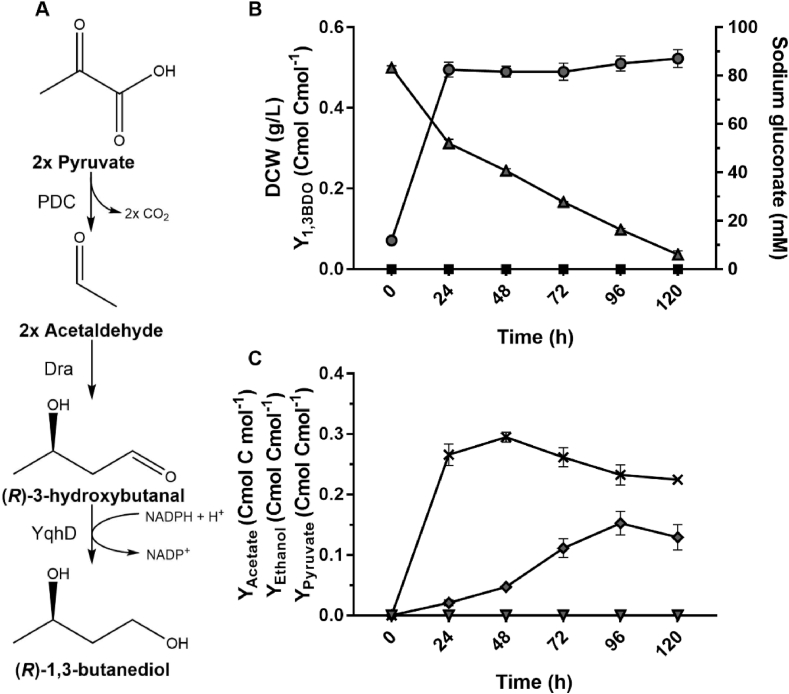
Evaluation of pyruvate-dependent biosynthetic pathway in *C. necator* H16ΔC_p306. (A) Schematic of pyruvate-dependent biosynthetic pathway consisting of pyruvate decarboxylase PDC, deoxyribose-5-phosphate aldolase Dra and aldehyde reductase YqhD. The bacteria DCW (circles), sodium gluconate concentration (triangles) and *(R)*-1,3-BDO yield Cmol Cmol^−1^ of sodium gluconate (squares) are presented in (B). (C) The yield (Cmol Cmol^−1^ of sodium gluconate) of major by-products excreted by the engineered *C. necator* H16 are highlighted as following: pyruvate (upside down triangles), acetate (crosses) and ethanol (diamonds). Strain H16ΔC-p306 was cultivated in NLMM supplemented with 2% (w/v) sodium gluconate and biosynthetic pathway gene expression was induced by addition of 0.01% (w/v) arabinose. Results represent the average of three biological replicates and error bars show standard deviation.

The pyruvate-dependent biosynthetic pathway has been recently developed for *(R)*-1,3-BDO production in *E. coli* (Kim et al., 2017; Nemr et al., 2018). This pathway consisting of pyruvate decarboxylase (PDC), deoxyribose-5-phosphate aldolase (Dra) and aldo/keto reductase (AKR) enables to convert pyruvate to *(R)*-1,3-BDO through acetaldehyde and (*R*)-3HBA intermediates. To utilise the pyruvate that accumulates in *C. necator* Δ*phaC* strains, the pyruvate-dependent pathway was implemented in this study. With YqhD_Ec_ proved suitable for conversion of (*R*)-3HBA to *(R)*-1,3-BDO in engineered *C. necator*, the gene of this enzyme was combined with *PDC* from *Z. mobilis* ZM4 and *dra* from *B. halodurans* into the plasmid pJLG306 yielding strain H16ΔC_p306 (Fig. 6A). However, similarly to H16ΔC-p26, this strain did not produce detectable quantities of *(R)*-1,3-BDO by HPLC-RI analysis under heterotrophic growth conditions (Fig. 6B). The further metabolite analysis revealed no accumulation of pyruvate, indicating that it is completely converted into acetaldehyde by PDC (Fig. 6C). However, high yields of acetate and ethanol suggest that Dra is ineffective in converting acetaldehyde to (*R*)-3HBA and causes a bottleneck in the pyruvate-dependent biosynthetic pathway. This is also supported by previous results showing that the gene copy number and expression level of *dra* contribute to the increase of *(R)*-1,3-BDO yield (Nemr et al., 2018). Furthermore, a rapid acetate synthesis from acetaldehyde is likely to be associated with acetaldehyde dehydrogenase AcoD activity in *C. necator* H16 (Priefert et al., 1992), whereas a low affinity of YqhD_Ec_ towards acetaldehyde (Pérez et al., 2008) can contribute to the gradual increase in the ethanol yield during the 120-h fermentation.

#### 3.4.2 Combining (*R*)-3HBCoA- and pyruvate-dependent pathways

Considering the absence of any detectable *(R)*-1,3-BDO production by the pyruvate-dependent pathway in *C. necator* and aiming to reduce accumulation of pyruvate and improve carbon flux through acetyl-CoA node, it was reasoned that the combination of both, pyruvate- and (*R*)-3HBCoA-dependent pathways, may improve *(R)*-1,3-BDO biosynthesis. As postulated previously, the acetoin dehydrogenase bypass can counteract an accumulation of pyruvate and utilise acetaldehyde that is generated as pyruvate-dependent pathway intermediate, by offering an alternative route to acetyl-CoA, especially, when pyruvate dehydrogenase complex is inhibited by elevated concentration of acetyl-CoA (Raberg et al., 2014).

**Fig. 7:**
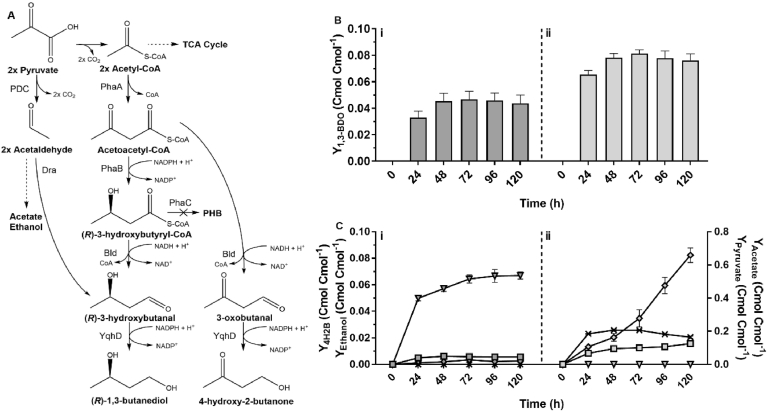
Improvement of *(R)*-1,3-BDO yield by combining 3-HBCoA-dependent and pyruvate-dependent pathways. (A) Schematic of cumulative biosynthetic pathway indicating routes of *(R)*-1,3-BDO and by-product formation. Batch fermentation product yields (Cmol Cmol^−1^ of sodium gluconate) for strains H16ΔC-p2 (i) and H16ΔC-p304 (ii) are presented as following: *(R)*-1,3-BDO (solid bars) (B); and 4H2B (squares), pyruvate (upside down triangles), acetate (crosses) and ethanol (diamonds) (C). Engineered strains were cultivated in NLMM supplemented with 2% (w/v) sodium gluconate and biosynthetic pathway gene expression was induced by addition of 0.01% (w/v) arabinose. Results represent the average of three biological replicates and error bars show standard deviation.

To combine (*R*)-3HBCoA- and pyruvate-dependent pathways, genes *bld, yqhD*_*Ec*_*, dra* and *PDC* were assembled into a plasmid pJLG304. *C. necator* strains H16ΔC_p304, harbouring pJLG304, and H16ΔC_p2, containing the 3-HBCoA-dependent pathway only, were compared for *(R)*-1,3-BDO and other major metabolite yields (Fig. 7; Supplementary Table 4). Strain H16ΔC_p304 showed 1.7-fold increase in *(R)*-1,3-BDO yield compared to H16ΔC_p2. Notably, similarly to H16ΔC_p306, no accumulation of pyruvate and high yields of acetate and ethanol were observed for strain H16ΔC_p304. Moreover, metabolite profiles vary considerably in an oxygen rich environment, with increased acetate yields by strain H16ΔC_p304 rising from 0.046 ± 0.002 to 0.206 ± 0.009 Cmol Cmol^−1^, after 72-h induction.

### 3.5 Engineering stable expression of *(R)-*1,3-BDO pathway genes

#### 3.5.1 Chromosomal integration of biosynthetic pathway

To improve genetic stability ensuring stable expression of *(R)-*1,3-BDO biosynthetic pathway genes, chromosomal integration of the constructs into *phaCAB* loci was performed. Simultaneously, heterologous genes for either 3-HBCoA-dependent pathway or combining the 3-HBCoA-dependent and pyruvate-dependent pathways were introduced. To ensure tuneable expression of chromosomally integrated heterologous genes, an arabinose inducible system (*araC*/P_*araBAD*_*)* preceded with terminator was integrated into the *phaC* locus upstream of either *bld* and *yqhD*_*Ec*_ (strain H16Δ1::54) or *bld*, *yqhD*_*Ec*_, *dra*, and *PDC* (strain H16Δ1::56). By design, utilisation of the *phaC* locus as a target integration site not only abolished the PHB synthesis but also ensured a controllable expression of *phaA* and *phaB*, which are required for *(R)-*1,3-BDO production. Nonetheless, engineered strains contained only a single copy of chromosomally integrated biosynthetic pathway genes, and despite a significant reduction in gene copy number comparing to the plasmid-based expression system, a detectable level of *(R)-*1,3-BDO was observed for both strains H16Δ1::54 and H16Δ1::56 under non-optimal growth conditions with limited aeration (Table 3).

**Table 3:** *(R)-*1,3-BDO and by-product yields in engineered *C. necator* strains. Cells were grown in 10 mL of 2 nitrogen-limiting minimal media supplemented with 2% sodium gluconate and 0.1% arabinose for 72 h.

Strain	Y_1,3BDO_ (Cmol Cmol^−1^)	Y_4H2B_ (Cmol Cmol^−1^)	Y_Acetate_ (Cmol Cmol^−1^)	Y_Ethanol_ (Cmol Cmol^−1^)	Y_Pyruvate_ (Cmol Cmol^−1^)
H16Δ1::54	0.008 ± 0.000	N.D.	0.022 ± 0.000	0.008 ± 0.000	0.345 ± 0.005
H16Δ1::54/Δ3::58	0.010 ± 0.001	N.D.	0.014 ± 0.002	0.013 ± 0.004	0.289 ± 0.023
H16Δ1::56	0.012 ± 0.001	N.D.	0.182 ± 0.010	0.108 ± 0.012	N.D.
H16Δ1::56/Δ3::58	0.017 ± 0.002	0.001 ± 0.000	0.136 ± 0.012	0.104 ± 0.003	0.016 ± 0.008
H16Δ1::56/Δ3::60	0.021 ± 0.001	0.001 ± 0.001	0.154 ± 0.012	0.110 ± 0.014	0.010 ± 0.005

Earlier results indicated that limited expression of either *bld* or *dra* can create a bottleneck in the *(R)-*1,3-BDO biosynthetic pathways. Moreover, *bld* from *C. saccharoperbutylacetonicum* is potentially an oxygen-sensitive enzyme, similarly to its homologue from *C. beijerinckii* (Yan and Chen, 1990). Therefore, to further improve strains H16Δ1::54 and H16Δ1::56, a second copy of these genes was introduced by replacing *sucCD*, deletion of which was identified in this study as beneficial for improving *(R)-*1,3-BDO yield. An additional copy of *bld* was integrated into the strains containing either the 3HBCoA-dependent pathway (H16Δ1::54/Δ3::58) or the combined 3HBCoA- and pyruvate-dependent pathway (H16Δ1::56/Δ3::58). The *bld* gene was placed under the control of a strong constitutive promoter (*P*_*8*_) (Alagesan et al., 2018a). The same strategy was employed for integration *bld* and *dra* into the strain with combined 3HBCoA-and pyruvate-dependent pathway (H16Δ1::56/Δ3::60). All engineered strains were screened by measuring *(R)-*1,3-BDO and by-products yields (Table 3). As expected, for all strains, diol yield was reduced compared with plasmid-based expression system. Nonetheless, a clear improvement of *(R)-*1,3-BDO biosynthesis was achieved by introducing additional copies of *dra* and/or *bld*.

#### 3.5.2 *(R)-*1,3-BDO production from CO_2_

**Fig. 8:**
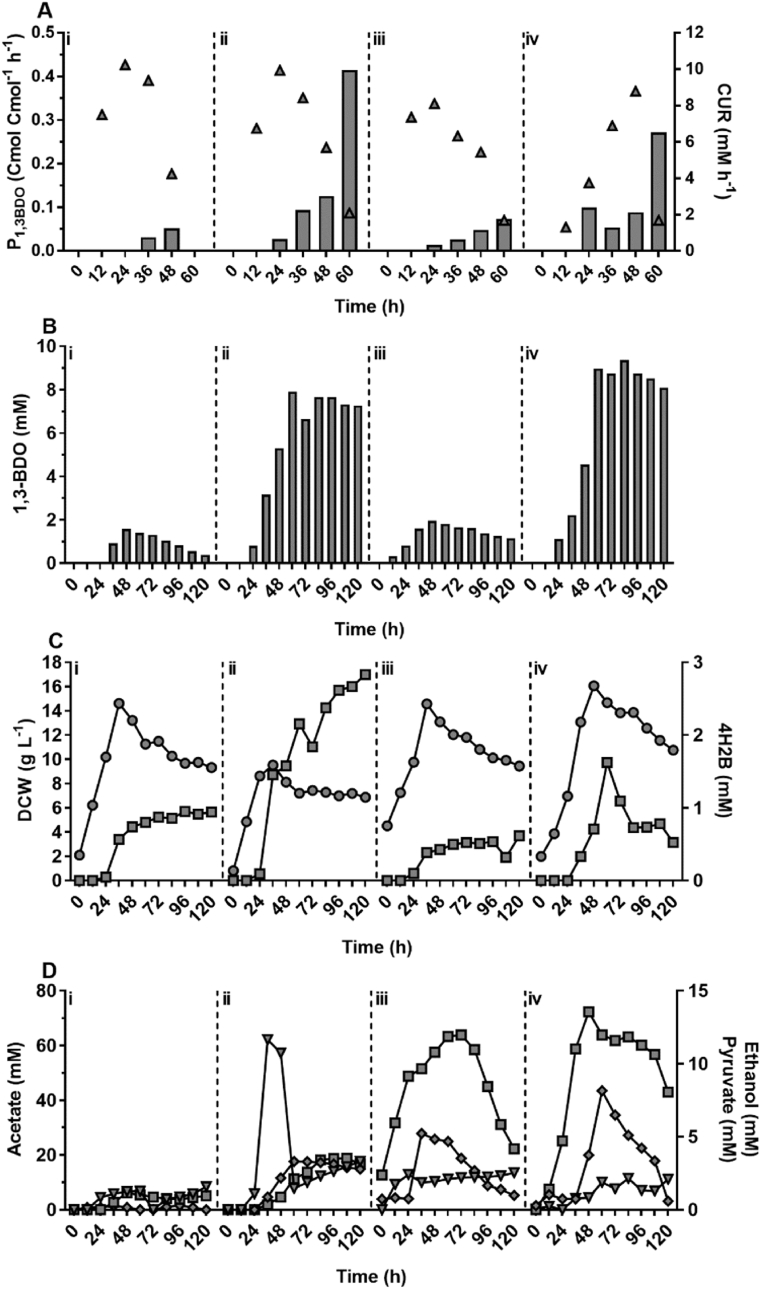
Autotrophic fed-batch fermentation of CO_2_ for (*R*)-1,3-BDO production using DASGIP parallel bioreactor system. Data for strains H16Δ1::54 (i), H16Δ1::54/Δ3::58 (ii), H16Δ1::56 (iii) and H16Δ1::56/Δ3::60 (iv) represented as following: production rate of *(R)-*1,3-BDO (solid bars) and CUR (triangles) (A); *(R)-*1,3-BDO titer (solid bars) (B); 4H2B yield (squares) and DCW (circles) (C); and acetate (squares), ethanol (diamonds), and pyruvate (upside down triangles) yields (D). Due to the continuous supply of carbon source, metabolite Cmol Cmol^−1^ yields were calculated by dividing metabolite production within a 12 h time period by average carbon uptake rate (CUR mmol h^−1^) for the identical 12-h time period. Results represent the average of three technical replicates (sampling) that were taken from single reactor for each strain.

With *C. necator* H16 capable of using CO_2_ as sole carbon source, autotrophic fed-batch fermentation was undertaken for production of *(R)-*1,3-BDO. Strains H16Δ1::54, H16Δ1::54/Δ3::58, H16Δ1::56 and H16Δ1::56/Δ3::60 were cultivated in 1.2 L bioreactors with a working volume of 750 mL, variable impeller agitation speed and a constant supply of CO_2_, H_2_ and air in the presence of 0.1% (w/v) arabinose (Fig. 8). As observed for metabolite profiling under heterotrophic growth conditions, increased availability of key pathway enzymes, namely Bld and Dra, considerably improved the *(R)-*1,3-BDO production when utilising the 3HBCoA-dependent pathway (strain H16Δ1::54/Δ3::58) and combination of 3HBCoA- and pyruvate-dependent pathways (H16Δ1::56/Δ3::60). For these strains, Maximum production rates of 0.41 and 0.27 Cmol Cmol^−1^ h^−1^ and titres of 7.8 and 9.5 mM, respectively, were measured in the early stationary phase (48–60 h). Despite a continuous supply of CO_2_ at this stage, cells were entering stationary phase due to the complete consumption of key elements such as nitrogen and/or phosphate, resulting in carbon flux being re-directed from biomass towards the *(R)-*1,3-BDO biosynthesis.

#### 3.5.3 Further improvement of autotrophic *(R)-*1,3-BDO production by increasing *bld* copy number

Moreover, to further evaluate if the increase in the copy number of biosynthetic pathways genes can improve *(R)-*1,3-BDO production, strains containing chromosomally integrated *bld*, *yqhD*_*Ec*_, *dra*, and *PDC*, were transformed with plasmid carrying *bld* and *dra* copies. *(R)-*1,3-BDO and by-product profiles of resulting strains H16Δ1::54_p14 (chromosomal *bld* and *yqhD*_*Ec*_; plasmid *bld*), H16Δ1::56_p14 (chromosomal *bld*, *yqhD*_*Ec*_, *dra*, and *PDC*; plasmid *bld*) and H16Δ1::56_p45 (chromosomal *bld*, *yqhD*_*Ec*_, *dra*, and *PDC*; plasmid *bld* and *dra*) were compared to earlier characterised strains H16ΔC_p2 and H16ΔC_p304 (Supplementary Figure 5). A significant improvement of *(R)-*1,3-BDO yield was observed in strains (H16Δ1::54_p14 and H16Δ1::56_p14) with additional copy of *bld* on the plasmid. Whereas, the addition of *dra* had only a marginal effect on the *(R)-*1,3-BDO yield. As observed previously, by introducing the pyruvate-dependent pathway, no pyruvate accumulation is observed demonstrating efficient metabolism of pyruvate to acetaldehyde facilitated by PDC.

**Fig. 9:**
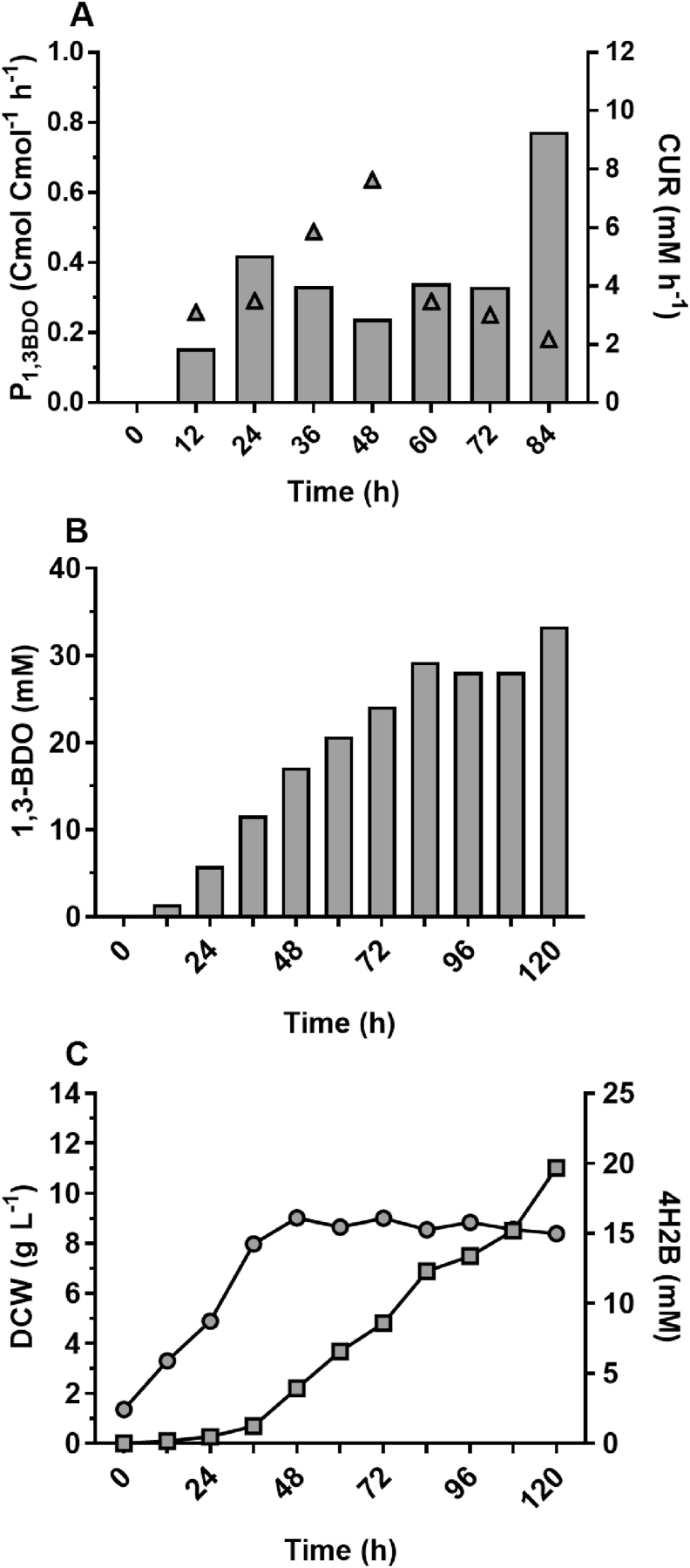
Autotrophic fed-batch fermentation of CO_2_ for (*R*)-1,3-BDO production using strain H16Δ1::56-p14. Data were obtained from single reactor and represented as following: production rate of *(R)-*1,3-BDO (solid bars) and CUR (triangles) (A); *(R)-*1,3-BDO titer (solid bars) (B); and 4H2B titer (squares) and DCW (circles) (C). Pyruvate, acetate and ethanol were not detected by HPLC analysis. Sampling was performed and Cmol Cmol^−1^ yields were calculated as described for Fig. 8.

Highest producing strains H16Δ1::56_p14 and H16Δ1::56_p45 were subjected to autotrophic fermentation using CO_2_ as a sole carbon source. Despite successful production of *(R)-*1,3-BDO in shake-flask mode, strain H16Δ1::56_p45 was genetically unstable due to the plasmid pJLG45 loss, which was observed at the early stage of fermentation by plating cell culture on non-selective medium and selective medium with chloramphenicol antibiotic. Therefore, the *(R)-*1,3-BDO or another metabolite production was inconsistent and was not subjected to further analysis. Nonetheless, H16Δ1::56_p14 achieved the highest reported *(R)-*1,3-BDO titre of 33 mmol L^−1^ (2.97 g/L) (Fig. 9). With theoretical yield of 1.00 for *(R)-*1,3-BDO production from CO_2_, a yield of 0.77 Cmol Cmol^−1^ for 72- to 84-h fermentation period and average yield of 0.4 Cmol Cmol^−1^ were obtained. Furthermore, 4H2B production was high (19.7 mmol L^−1^ titer and average yield close to 0.3). 4H2B yield increased during later stage of fermentation indicating insufficient conversion of acetoacetyl-CoA to 3-hydroxybutanal facilitated by PhaB, despite being under the control of the arabinose inducible system. With such high yields of *(R)-*1,3-BDO and 4H2B there was no other by-products detectable.

## 4 Conclusions

Here, we report the stepwise engineering of *C. necator* H16 for production of (*R*)-1,3-BDO from CO_2_. To achieve this, two alternative heterologous (*R*)-1,3-BDO biosynthetic pathways, based on utilisation of either (*R*)-3HBCoA or pyruvate as precursors, were investigated. Initially, the (*R*)-1,3-BDO biosynthesis was achieved by heterologous gene expression of either *C. saccharoperbutylacetonicum bld* in combination with *E. coli yqhD* or *C. acetobutylicum adhE2*. The (*R*)-1,3-BDO yield was improved through the genetic inactivation of the PHB biosynthesis by deletion of either *phaC1* gene or *phaCAB* operon and redirecting excess carbon toward the diol production. (*R*)-1,3-BDO-producing strains were further improved by introducing extra copies of *phaA, phaB1*, *bld* and *dra*, as well as by deleting *sucCD* genes. An alternative (*R*)-1,3-BDO biosynthetic pathway was implemented by heterologous expression of *PDC* from *Z. mobilis*, and *dra* and *yqhD* from *E. coli*. The introduction of this biosynthetic pathway did not yield a detectable level of (*R*)-1,3-BDO, whereas the combination of both biosynthetic pathways resulted in a highest diol production. Further to this, genes of both (*R*)-1,3-BDO biosynthetic pathways were chromosomally integrated ensuring the genetic stability of engineered strains. Application of (*R*)-3HBCoA- and pyruvate-dependent pathways, in combination with abolishing the PHB biosynthesis and reducing the flux through the tricarboxylic acid cycle, enabled to engineer a strain that was able to produce more than 2.97 g/L of (*R*)-1,3-BDO *via* autotrophic fermentation from CO_2_. In this fermentation mode a large proportion of carbon (up to 40% Cmol Cmol^−1^) was directed to the (*R*)-1,3-BDO. In conclusion, this study demonstrates that engineered *C. necator* H16 can be effectively utilised for diol production.
