# Supplementary material for: Engineering Cupriavidus necator H16 for the autotrophic production of (R)-1,3-butanediol
Source: Metab Eng. 2021 Sep;67:262–76. doi: 10.1016/j.ymben.2021.06.010 (PMC8449065; doi:10.1016/j.ymben.2021.06.010)
Supplement: Multimedia component 1 [file mmc1.docx]

**Supplementary Information**

Engineering *Cupriavidus necator* H16 for the autotrophic production of (*R*)-1,3-butanediol

Joshua Luke Gascoyne, Rajesh Reddy Bommareddy, Stephan Heeb, Naglis Malys^*^

BBSRC/EPSRC Synthetic Biology Research Centre (SBRC), School of Life Sciences, Biodiscovery Institute, The University of Nottingham, Nottingham, NG7 2RD, United Kingdom

^*^Corresponding author: e-mail address: n.malys@gmail.com

**Supplementary Methods**

**1. Plasmid construction**

For construction of plasmids, two modular plasmids pJLG1 and pJLG2 containing 1,3-BDO biosynthetic pathway variants were assembled using the USER cloning method as described previously (Bitinaite et al., 2007). USER oligonucleotides were designed to contain one deoxyuridine (dU) residue 6–8 nucleotides from the 5’ end with complimentary overlap sequences. Genes to be assembled were amplified by PCR using Phusion U polymerase and genomic or plasmid DNA as a template (Invitrogen, CA, USA). The plasmid pBBR1-USER (Alagesan et al., 2018) vector was prepared by digesting with XbaI followed by nicking with Nt.BbvCI. PCR fragments were assembled into pBBR1-2USER vector using USER enzyme according to manufacturer’s recommendations (New England Biolabs). Both modular plasmids pJLG1 and pJLG2 contain a pBBR1 origin of replication, kanamycin resistance gene, arabinose inducible system araC/*P_araBAD_* and several unique restriction sites flanking gene sequences and forming the modular arrangement as following: restriction sites XbaI and EcoRI flanking the arabinose inducible system; EcoRI and NdeI sites flanking RBS 1; NdeI and SpeI sites flanking gene 1; an SpeI site upstream of RBS 2 and gene 2 followed by adjacent XhoI and NheI sites, allowing rapid assembly of new constructs. Further details how each plasmid was assembled are provided below:

pJLG1: arabinose inducible system araC/*P_araBAD_*, amplified from pEH006 (Hanko et al., 2017) using oligonucleotide primers N1_u_ara_f and N1_u_ara_r, and *adhE1* (CA_P0162), amplified using oligonucleotide primers PJG15_U_Adhe_F and PJG16_U_Adhe_R from template DNA synthesised and codon optimised by GeneArt Gene Synthesis (Thermo Fisher Scientific), were assembled into pBBR1-2USER vector using USER cloning method as described above.

pJLG2: arabinose inducible system araC/*P_araBAD_*, amplified from pEH006 (Hanko et al., 2017) using oligonucleotide primers N1_u_ara_f and N1_u_ara_r, *bld* (AAP42563), amplified with oligonucleotide primers PJG17_U_Bld_F and PJG18_U_Bld_R from template DNA synthesised and codon optimised by GeneArt Gene Synthesis (Thermo Fisher Scientific), and *yqhD* (b3011) amplified with oligonucleotide primers PJG9_U_YqhD_F and PJG10_U_YqhD_R from *E. coli* MG1655 genomic DNA, were assembled into pBBR1-2USER vector using USER cloning method as described above.

pJLG3: *s-adh* (WP_077844196) was amplified with PJG7_U_sAdh_F and PJG8_U_sAdh_R from template DNA synthesised and codon optimised by Gene Art Gene Synthesis. The PCR product was cloned into pJLG2 using restriction sites SpeI and XhoI.

pJLG4: *hibadh* (H16_RS24705) was amplified with PJG13_U_HIB_F and PJG14_U_HIB_R from purified *C. necator* H16 genomic DNA. The PCR product was cloned into pJLG2 using restriction sites SpeI and XhoI.

pJLG5: *gbD* (H16_RS07715) was amplified with PJG11_U_gbd_F and PJG12_U_gbd_R from purified *C. necator* H16 genomic DNA. The PCR product was cloned into pJLG2 using restriction sites SpeI and XhoI.

pJLG11: *phaA* and *phaB1* (H16_A1438 and H16_A1439) were amplified as one fragment with native ribosomal binding sites by P20_phaA_Xho_F and P21_phaB1_NheI_R from purified *C. necator* H16 genomic DNA. The PCR product was cloned into pJLG2 using restriction sites XhoI and NheI.

pJLG14: *bld* (AAP42563) extracted from pJLG2 was cloned into pJLG1 using restriction sites EcoRI and SpeI.

pJLG15: *yqhD* (PP_2492) was amplified with P25_YqhD.PP_SpeI_F and P26_YqhD.PP_XhoI_R from purified *P. putida* KT2440 purified genomic DNA. The PCR product was cloned into pJLG2 using restriction sites XhoI and NheI.

pJLG16: *yvgN* (BSU33400) was amplified with P27_YvgN_SpeI_F and P28_YvgN_XhoI_R from purified *B. subtilius* 168 purified genomic DNA. The PCR product was cloned into pJLG2 using restriction sites XhoI and NheI.

pJLG17: *yhdN* (BSU09530) was amplified with P29_YhdN_SpeI_F and P30_YhdN_XhoI_R from purified *B. subtilius* 168 purified genomic DNA. The PCR product was cloned into pJLG2 using restriction sites XhoI and NheI.

pJLG18: *ydjG* (b1771) was amplified with P31_ydjG_SpeI_F and P32_ydjG_XhoI_R from purified *E. coli* MG1655 genomic DNA. The PCR product was cloned into pJLG2 using restriction sites XhoI and NheI.

pJLG19: *gpr* (b3001) was amplified with P33_gpr_SpeI_F and P32_ydjG_XhoI_R from purified *E. coli* MG1655 genomic DNA. The PCR product was cloned into pJLG2 using restriction sites XhoI and NheI.

pJLG20: aldo-keto reductase PA_1127 was amplified with P35_ARK.PA_SpeI_F and P36_AKR.PA_XhoI_R from purified *P. aeruginosa* PAO1 genomic DNA. The PCR product was cloned into pJLG2 using restriction sites XhoI and NheI.

pJLG21: 1,3-propanediol dehydrogenase BMD_1654 was amplified with P37_PDOD1_SpeI_F and P38_PDOD1_XhoI_R from purified *B. megaterium*  DSM319 genomic DNA. The PCR product was cloned into pJLG2 using restriction sites XhoI and NheI.

pJLG22: 1,3-propanediol dehydrogenase BMD_2640 was amplified with P39_PDOD2_SpeI_F and P40_PDOD2_XhoI_R from purified *B. megaterium*  DSM319 genomic DNA. The PCR product was cloned into pJLG2 using restriction sites XhoI and NheI.

pJLG23: *eutE* (BMD_3362) was amplified with P43_eutE.BMD_EcoRI_F and P44_eutE.BMD_SpeI_R from purified *B. megaterium* DSM319 genomic DNA. The PCR product was cloned into pJLG2 using restriction sites EcoRI and SpeI.

pJLG24: *eutE* (b2455) was amplified with P41_eutE.EC_EcoRI_F and P42_eutE.EC_SpeI_R from purified *E. coli* MG1655 genomic DNA. The PCR product was cloned into pJLG2 using restriction sites EcoRI and SpeI.

pJLG25: *adhE* (b1241) was amplified with P45_adhE.EC_NdeI_F and P46_adhE.EC_SpeI_R from purified *E. coli* MG1655 genomic DNA. The PCR product was cloned into pJLG1 using restriction sites NdeI and SpeI.

pJLG26: *adhE2* (CA_P0035) was amplified with P45_adhE.EC_NdeI_F and P46_adhE.EC_SpeI_R from purified *C. acetobutylicum* ATCC 824 genomic DNA. The PCR product was cloned into pJLG1 using restriction sites NdeI and SpeI.

pJLG27: *yqhD* (b3011) was amplified with pJLG27_yqhD.ECHiFi_F and pJLG27_yqhD.ECHiFI_R and *bld* (AAP42563) with pJLG27_bldHiFi_F and pJLG27_bldHiFi_R from pJLG2 and assembled using the NEBuilder HiFi DNA Assembly Master Mix into the pJLG2 backbone digested with EcoRI and XhoI.

pJLG35: the *phaA* and *phaB* fragment was extracted from pJLG11 and cloned into pJLG15 using restriction sites XhoI and NheI.

pJLG36: the *bktB* and *phaB* fragment was extracted from pJLG12 and cloned into pJLG15 using restriction sites XhoI and NheI.

pJLG37: *yqhD* (b3011) was amplified with 37_yqhD.EC_EcoRI_F and 37_yqhD.EC_SpeI_R from pJLG2. The PCR product was cloned into pJLG1 using restriction sites NdeI and SpeI.

pJLG38: the *phaA* and *phaB* fragment was extracted from pJLG11 and cloned into pJLG20 using restriction sites XhoI and NheI.

pJLG44: the *dra* and *pdc* fragment was extracted from synthetic plasmid and cloned into pJLG2 using restriction sites XhoI and NheI

pJLG45: *dra* (BH1351) was amplified with 45.dra_Spe_F and 45.dra_Xho_R from template DNA synthesised and codon optimised by Gene Art Gene Synthesis. The PCR product was cloned into pJLG14 using restriction sites SpeI and XhoI.

pJLG46: the *yqhD, dra* and *pdc* operon was amplified from pJLG44 with 27.yqhD.EC_F and 303.PDCOpt_Nhe_R and cloned into pJLG401 using restriction sites NdeI and NheI.

pJLG47: *phaB1* (H16_A1439) was amplified with native ribosomal binding site by 44.phaB1_Xho_F and 44.phaB1_Nhe_R from purified *C. necator* H16 genomic DNA. The PCR product was cloned into pJLG2 using restriction sites XhoI and NheI.

pJLG304: the *yqhD, dra* and *pdc* operon was amplified from pJLG44 with 27.yqhD.EC_F and 303.PDCOpt_Nhe_R and cloned into pJLG1 using restriction sites NdeI and NheI.

pJLG306: *phaB1* (H16_A1439) was amplified with native ribosomal binding site by 44.phaB1_Xho_F and 44.phaB1_Nhe_R from purified *C. necator* H16 genomic DNA. The PCR product was cloned into pJLG2 using restriction sites XhoI and NheI.

The pLO3 suicide vector was utilised for all genomic manipulations of *C. necator.* With homologous recombination the primary method used for genomic manipulations, homologous chromosomal regions upstream and downstream of the desired genomic manipulation were amplified through PCR with complementary regions resulting in PCR fragment fusion through SOEing PCR or through HiFi assembly into the multiple cloning site of the pLO3 backbone.

pJLG50: homology arms upstream and downstream of *phaB1* were amplified with phaB1_Sac_UP_F, phaB1_OVL_R and phaB1_OVL_F, phaB1_Pac_DN_R, respectively. PCR products were then fused together using SOEing PCR with primers phaB1_Sac_UP_F and phaB1_Pac_DN_R. The fusion PCR product was cloned into pLO3 using restriction sites SacI and PacI.

pJLG51: homology arms upstream and downstream of *iclA* were amplified with iclA_Sac_UP_F, iclA_OVL_R and iclA_OVL_F, iclA_Pac_DN_R, respectively. PCR products were then fused together using SOEing PCR with primers iclA_Sac_UP_F and iclA_Pac_DN_R. The fusion PCR product was cloned into pLO3 using restriction sites SacI and PacI.

pJLG52: homology arms upstream and downstream of *iclB* were amplified with iclB_Sac_UP_F, iclB_OVL_R and iclB_OVL_F, iclB_Pac_DN_R, respectively. PCR products were then fused together using SOEing PCR with primers iclB_Sac_UP_F and iclB_Pac_DN_R. The fusion PCR product was cloned into pLO3 using restriction sites SacI and PacI.

pJLG53: homology arms upstream of *sucC* and downstream of *sucD* were amplified with sucC_Sac_UP_F, sucC_OVL_R and sucD_OVL_F, sucD_Pac_DN_R, respectively. PCR products were then fused together using SOEing PCR with primers sucC_Sac_UP_F and sucD_Pac_DN_R. The fusion PCR product was cloned into pLO3 using restriction sites SacI and PacI.

pJLG54: homology arms upstream and downstream of *phaC* were amplified with phaC_750Up_SacI_F, phaC_OVL_R and phaC_OVL_F, phaC_750DN_PmeI_R, respectively from purified *C. necator* H16 genomic DNA. PCR products were then fused together using SOEing PCR with primers phaC_750Up_SacI_F and phaC_750DN_PmeI_R. The fusion PCR product was cloned into pLO3 using restriction sites SacI and PmeI creating the *phaC* deletion empty vector pLO3_phaC::e. The araC/*P_araBAD_*, *bld* and *yqhD_Ec_* operon was amplified from pJLG2 with pLO3_pJLGPacT_F and pLO3_pJLGBsu36I_R. The PCR product was cloned into pLO3-phaC::e using restriction sites PacI and PmeI..

pJLG56: The araC/*P_araBAD_*, *bld*, *yqhD_Ec_*, *dra* and *PDC* operon was amplified from pJLG444 with pLO3_pJLGPacT_F and pLO3_pJLGBsu36I_R. The PCR product was cloned into pLO3-phaC::e using restriction sites PacI and PmeI.

pJLG57: The araC/*P_araBAD_*, *yqhD_Ec_*, *dra* and *PDC* operon was amplified from pJLG446 with pLO3_pJLGPacT_F and pLO3_pJLGBsu36I_R. The PCR product was cloned into pLO3-phaC::e using restriction sites PacI and PmeI.

pJLG58: assembly was undertaken using the NEBuilder Hi Fi DNA Assembly Master Mix. Homology arms upstream of *sucC* and downstream of *sucD* were amplified with sucC_UP.HiFi_F, sucC_UP.HiFi_R and sucD_DN.HiFi_F, sucD_DN.HiFi_R, respectively from purified *C. necator* H16 genomic DNA. The constitutive *P_8_* promoter was amplified with P8.HiFi_F and P8.HiFi_R from pBBR1MCS-2-*P_8_* (Alagesan et al., 2018)and *bld* (AAP42563) amplified with bld.HiFi_F and bld.HiFi_R from template DNA synthesised and codon optimised by Gene Art Gene Synthesis or pJLG2. The PCR products were then assembled into pLO3 backbone linearized with SacI and XbaI. Terminators were also placed directly upstream of the *P_8_* promoter and downstream of *bld* (AAP42563).

pJLG59: *dra* (BH1351) was amplified with 45.dra_Spe_F and 45.dra_Xho_R from template DNA synthesised and codon optimised by GeneArt Gene Synthesis. The PCR product was cloned into pJLG58 using restriction sites SpeI and XhoI.

.

**Supplementary Tables and Figures**

**Supplementary Table 1.** Plasmids used in this study.

| **Plasmid** | **Description** | **Source** |
| --- | --- | --- |
| pBBR1-2USER | host plasmid with Km^r^ and pBBR1 ori | (Alagesan et al., 2018) |
| pJLG1 | pBBR1-USER with *P_araBAD_*, a*dhE1** | This work |
| pJLG2 | pBBR1-USER with *P_araBAD_*, *bld**, y*qhD* (b3011) | This work |
| pJLG3 | pBBR1-USER with *P_araBAD_, bld*, s-adh** | This work |
| pJLG4 | pBBR1-USER with *P_araBAD_*, *bld*, HIBADH* | This work |
| pJLG5 | pBBR1-USER with *P_araBAD_*, *bld*, gbd* | This work |
| pJLG11 | pBBR1-USER with *P_araBAD_, bld*, yqhD* ( b3011), *phaA, phaB1* | This work |
| pJLG14 | pBBR1-USER with *P_araBAD_, bld** | This work |
| pJLG15 | pBBR1-USER with *P_araBAD_, bld*, yqhD* (PP_2492) | This work |
| pJLG16 | pBBR1-USER with *P_araBAD_, bld*, yvgN* | This work |
| pJLG17 | pBBR1-USER with *P_araBAD_, bld*, yhdN* | This work |
| pJLG18 | pBBR1-USER with *P_araBAD_, bld*, ydjG* | This work |
| pJLG19 | pBBR1-USER with *P_araBAD_, bld*, gpr* | This work |
| pJLG20 | pBBR1-USER with *P_araBAD_, bld*,* PA_1127 | This work |
| pJLG21 | pBBR1-USER with *P_araBAD_, bld*,* BMD_1654 | This work |
| pJLG22 | pBBR1-USER with *P_araBAD_, bld*,* BMD_2640 | This wo/rk |
| pJLG23 | pBBR1-USER with *P_araBAD_, eutE* (BMD_3362), y*qhD* (b3011) | This work |
| pJLG24 | pBBR1-USER with *P_araBAD_, eutE* (b2455), y*qhD* (b3011) | This work |
| pJLG25 | pBBR1-USER with *P_araBAD_,* a*dhE* (b1241) | This work |
| pJLG26 | pBBR1-USER with *P_araBAD_*, a*dhE2* | This work |
| pJLG27 | pBBR1-USER with *P_araBAD_, yqhD* (b3011), *bld** | This work |
| pJLG35 | pBBR1-USER with *P_araBAD_, bld*, yqhD* (PP_2492), *phaA, phaB1* | This work |
| pJLG36 | pBBR1-USER with *P_araBAD_, bld*, yqhD* (PP_2492), *bktB, phaB1* | This work |
| pJLG38 | pBBR1-USER with *P_araBAD_, bld*,* PA_1127, *phaA, phaB1* | This work |
| pJLG304 | pBBR1-USER with *P_araBAD_, bld**, y*qhD* (b3011), *dra**, *PDC** | This work |
| pJLG306 | pBBR1-USER with *P_araBAD_,*, y*qhD* (b3011), *dra**, *PDC** | This work |
| pJLG44 | pBBR1-USER with *P_araBAD_, bld**, y*qhD* (b3011), *phaB1* | This work |
| pJLG45 | pBBR1-USER with *P_araBAD_, bld**, *dra** | This work |
| pJLG46 | pBBR1-USER with *P_araBAD_,*, y*qhD* (b3011), *dra**, *pdc** | This work |
| pJLG47 | pBBR1-USER with *P_araBAD_, bld**, y*qhD* (b3011), *phaB1* | This work |
| pBBR1MCS-2-P8 | Source of *P_8_* promoter | (Alagesan et al., 2018) |
| pLO3 | host plasmid with Tet^R^,, *sacB*, RP4 transfer ori, pBR322 ori | (Lenz and Friedrich, 1998) |
| pJLG50 | pLO3, *phaB1* (H16_A1439) deletion vector | This work |
| pJLG51 | pLO3, *iclA* (H16_A2211) deletion vector | This work |
| pJLG52 | pLO3, *iclB* (H16_A2227) deletion vector | This work |
| pJLG53 | pLO3, *sucCD* (H16_A0547, H16_A0548) deletion vector | This work |
| pJLG54 | pLO3, *phaC1* (H16_A1437)::*P_araBAD_, bld*, yqhD* (b3011) operon deletion/integration vector | This work |
| pJLG56 | pLO3, *phaC1* (H16_A1437)::*P_araBAD_, bld*, yqhD* (b3011), *dra**, *PDC** operon deletion/integration vector | This work |
| pJLG57 | pLO3, *phaC1* (H16_A1437)::*P_araBAD_, yqhD* (b3011), *dra**, *PDC** operon deletion/integration vector | This work |
| pJLG58 | pLO3, *sucCD* (H16_A0547, H16_A0548)::*P_8_, bld** operon deletion/integration vector | This work |
| pJLG59 | pLO3, *sucCD* (H16_A0547, H16_A0548)::*P_8_, bld*, dra** operon deletion/integration vector | This work |

*denotes codon optimisation using *C. necator* H16 codon usage; Km^r^ - kanamycin resistance; Tet^R^ - tetracycline resistance.

**Supplementary Table 2.** Oligonucleotide primers used in this study. All oligonucleotide primers were synthesised by Sigma-Aldrich. Restriction endonuclease sites are underlined.

| Name | Sequence 5' to 3' |
| --- | --- |
| N1_u_ara_f | gggaaaguctagattatgacaacttgacggctac |
| N2_u_ara_r | atatctccutcttaaaagatcttttgaattccc |
| PJG7_U_sAdh_F | agttaagtauaagaaggagatataacatgaagggcttcgccatgc |
| PJG8_U_sAdh_R | ggagacauctcgagtcacaggatcaccacggc |
| PJG9_U_YqhD_F | agttaagtauaagaaggagatataacatgaacaactttaatctgcacaccc |
| PJG10_U_YqhD_R | ggagacauctcgaggcgtaaaaagcttagcgg |
| PJG11_U_gbd_F | agttaagtauaagaaggagatataacatggcgtttatctactatctgaccc |
| PJG12_U_gbd_R | ggagacauctcgagctacatggactgctcaagcatacg |
| PJG13_U_HIB_F | agttaagtauaagaaggagatataacatgcatatcgccttcatcggc |
| PJG14_U_HIB_R | ggagacauctcgagtcattgcttgccctccttgt |
| PJG15_U_Adhe_F | aggagatauacatatgaaggtgaccaccgtgaag |
| PJG16_U_Adhe_R | ggagacauagtctcgagactagttcagggctg |
| PJG17_U_Bld_F | aggagatauacatatgatcaaggacaccctggtg |
| PJG18_U_Bld_R | atacttaacuagtcgagactagttcagccgg |
| P20_phaA_Xho_F | atatctcgagcgcttgcatgagtgccgg |
| P21_phaB1_NheI_R | atatgctagccaggtcagcccatatgcaggc |
| P22_btkB_Xho_F | atatctcgagttaggtaaaagtacgctcgttcgatttcg |
| P23_btkB_SacI_R | atatgagctctaacctcagatacgctcgaagatggc |
| P24_phaB1_SacI_F | atatgagctcggaaggggttttccggggc |
| P25_YqhD.PP_SpeI_F | atatactagtaagaaggagatataatgctcaattttgacttccacaacc |
| P26_YqhD.PP_XhoI_R | tatactcgagttagaggctggcttcaagcaccctg |
| P27_YvgN_SpeI_F | atatactagtaagaaggagatataatgccaacaagtttaaaagatactgtaaagttacataacggagttg |
| P28_YvgN_XhoI_R | tatactcgagttaaaacagaagctcatcaggatttggaccgac |
| P29_YhdN_SpeI_F | atatactagtaagaaggagatataatggaatataccagtatagcagatacaggaatagaagcctc |
| P30_YhdN_XhoI_R | tatactcgagttatatttcctctctggtcggcgggg |
| P31_ydjG_SpeI_F | atatactagtaagaaggagatataatgaaaaagatacctttaggcacaacggatattacgc |
| P32_ydjG_XhoI_R | tatactcgagttaacgctccagggcctctgcc |
| P33_gpr_SpeI_F | atatactagtaagaaggagatataatggtctggttagcgaatcccgaacg |
| p34_gpr_XhoI_R | tatactcgagtcatttatcggaagacgcctgccacag |
| P35_ARK.PA_SpeI_F | atatactagtaagaaggagatataatgagcgttgaaagcattcgcatcgag |
| P36_AKR.PA_XhoI_R | tatactcgagtcaggcgttgcggctggg |
| P37_PDOD1_SpeI_F | atatactagtaagaaggagatataatgaaagggtattcaaaattttgtatgcctaactcagtcttttatgg |
| P38_PDOD1_XhoI_R | tatactcgagttaagattcaacagtatttttgcttcttgaaaaatcgtagttatagcagtaatag |
| P39_PDOD2_SpeI_F | atatactagtaagaaggagatataatgggaatttatgaactacttgtaccacgcac |
| P40_PDOD2_XhoI_R | tatactcgagttagcaggacttccgctttgaatcgca |
| P41_eutE.EC_EcoRI_F | atatgaattcaaaagatcttttaagaaggagatatacatatgaatcaacaggatattgaacaggtggtgaaagc |
| P42_eutE.EC_SpeI_R | tataactagtttaaacaatgcgaaacgcatcgactaatacacag |
| P43_eutE.BMD_EcoRI_F | atatgaattcaaaagatcttttaagaaggagatatacatatgcagtttgatcaagatcttcaatctttacaagagatgc |
| P44_eutE.BMD_SpeI_R | tataactagtttagtttgtttgatatttttgaagtgcttgattaaccatttcagaaattg |
| P45_adhE.EC_NdeI_F | atatcatatgatggctgttactaatgtcgctgaacttaacgc |
| P46_adhE.EC_SpeI_R | tataactagtttaagcggattttttcgcttttttctcagctttagc |
| P47_adhE2.CA_NdeI_F | atatcatatgatgaaagttacaaatcaaaaagaactaaaacaaaagctaaatgaattgagagaag |
| P48_adhE2.CA_SpeI_R | tataactagtttaaaatgattttatatagatatccttaagttcacttataagtggatacctaggattagctgttg |
| sucC_Sac_UP_F | atatgagctcgcgccattacggccgag |
| sucC_OVL_R | ggcgaggtacgacttacagcattgcgatattcatgcgtgtttcctttcgggtagg |
| sucD_OVL_F | cctacccgaaaggaaacacgcatgaatatcgcaatgctgtaagtcgtacctcgcc |
| sucD_Pac_DN_R | tatattaattaaaagcgctccatgaccttgagc |
| sucC_UP_F | gtggcgaggcctgatgcg |
| sucD_DN_R | aatctcgtcaggcggcctg |
| iclA_Sac_UP_F | atatgagctcgcgcctgctcttcatcgatgatcg |
| iclA_OVL_R | ggttggcgttttgcttggggtattactggtatcagaagggccatgggaatttcctaagaagaaag |
| iclA_OVL_F | ctttcttcttaggaaattcccatggccttctgataccagtaataccccaagcaaaacgccaacc |
| iclA_Pac_DN_R | tatattaattaacaaccatcgagcagcgagatgttc |
| iclA_UP_F | atcggctggatggcgcc |
| iclA_DN_R | ggcaggagtggtcacggg |
| iclB_Sac_UP_F | atatgagctccgccttccacctgccgtg |
| iclB_OVL_R | gggtgaccaggagagtcaggcggtcatggtcactctcctgatgatgc |
| iclB_OVL_F | gcatcatcaggagagtgaccatgaccgcctgactctcctggtcaccc |
| iclB_Pac_DN_R | tatattaattaacgtcgggcgtcttcagttc |
| iclB_UP_F | tcggccatgactggggttcg |
| iclB_DN_R | gggcgcagggtcaggc |
| pJLG27_yqhD.ECHiFi_F | cttttaagaaaggagatatagagctcatgaacaactttaatctgcacac |
| pJLG27_yqhD.ECHiFI_R | atatctccttcttactagtttagcgggcggcttc |
| pJLG27_bldHiFi_F | cgcccgctaaactagtaagaaggagatataatgatcaaggacaccctgg |
| pJLG27_bldHiFi_R | tagctgaggagacattagtctcgagtcagccggccagcac |
| phaC_750Up_SacI_F | tatagagctcccgacgccggtcgcttctactcctatc |
| phaC_OVL_R | ggcactcatgcaagcgtcatgcatacctcaggatactcgagatattaattaaatacgccatgatttgattgtctctctgccg |
| phaC_OVL_F | cggcagagagacaatcaaatcatggcgtatttaattaatatctcgagtatcctgaggtatgcatgacgcttgcatgagtgcc |
| phaC_750DN_PmeI_R | atatgtttaaacgcacgaactcgtcggtcttgaaggc |
| phaB_OVL_F | cggcagagagacaatcaaatcatggcgtatttaattaatatctcgagtatcctgaggtatggctgacctgccggcctg |
| phaB_750DN_Pme_R | atatgtttaaacatgttcttttccaggtaggtgcccatcatg |
| pLO3_pJLGPacT_F | tatattaattaaagaaggccatcctgacggatggc |
| pLO3_pJLGBsu36I_R | atatcctcaggcatttgagaagcacacgg |
| 37_yqhD.EC_EcoRI_F | tatagaattcaaaagatcttttaagaaggagatatacatatgaacaactttaatctgcacaccccaacc |
| 37_yqhD.EC_SpeI_R | atatactagtttagcgggcggcttcgtatatacgg |
| sucC_UP.HiFi_F | cacctagatccttttaattcgagctctcgcgttcggtcgtcggcaagatca |
| sucC_UP.HiFi_R | ttaatttggtaccttatgcgtgtttcctttcgggtaggcgtaaaagg |
| P8.HiFi_F | aaaggaaacacgcataaggtaccaaattaagcagaaggccatc |
| P8.HiFi_R | taaaagatcttttgaattctaagatgaatctattatatcgccg |
| bld.HiFi_F | catcttagaattcaaaagatcttttaagaatacatatgatcaaggacaccc |
| bld.HiFi_R | cgttttatttgatgctagcctcgagactagttcagccggccagc |
| sucD_DN.HiFi_F | aactagtctcgaggctagcatcaaataaaacgaaaggccttcgggcctttcgttttatctgttgtttaccggtgtcgtacctcgccattccagcgagcgctgg |
| sucD_DN.HiFi_R | ttaaacagtcgactctagacttaattaatcaggcggcctgtgccgaccggcgg |
| suC-1200UP_F | gctgtcgaaccagcgctttgc |
| sucD-1200DN_R | ctattgagcagtaaccgtgccagc |
| 44.phaB1_Xho_F | atatctcgagggaaggggttttccggggcc |
| 44.phaB1_Nhe_R | tatagctagcgctagccaggtcagcccatatgcag |

**Supplementary Table 3.** Maximum specific growth rate *C. necator* H16 in the presence of *(R)*-1,3-BDO.

| *(R)*-1,3-BDO (mM)^a^ | µ_max_ (h^-1^)^b^ |
| --- | --- |
| 554.8 | 0.161 ± 0.003 |
| 332.9 | 0.199 ± 0.008 |
| 166.4 | 0.247 ± 0.051 |
| 83.2 | 0.392 ± 0.062 |
| 22.2 | 0.308 ± 0.002 |
| 0 | 0.355 ± 0.007 |

^a^The impact of *(R)*-1,3-BDO on the growth of *C. necator* H16 was investigated by growing cells in minimal media supplemented with 0, 22.2, 83.2, 166.4, 332.9 and 554.8 mM *(R)*-1,3-BDO.

^b^Cell density was measured at regular 2-hour intervals and maximum specific growth rate (µ_max_) was calculated using Equation 3 as described in *Materials and Methods*.

**Supplementary Table 4.** Carbon mol per carbon mol yield of 1,3-BDO, 4H2B, acetate, ethanol and pyruvate in strains H16∆C-p2 and H16∆C-p304. Cells were cultivated for 72 h in 10 mL of NLMM supplemented with 2 % (w/v) sodium gluconate and 0.01 % (w/v) L-arabinose in 50 mL falcon tubes.

| **Strain** | **Y_1,3BDO_**  **(Cmol Cmol^-1^)** | **Y_4H2B_**  **(Cmol Cmol^-1^)** | **Y_Acetate_**  **(Cmol Cmol^-1^)** | **Y_Ethanol_**  **(Cmol Cmol^-1^)** | **Y_Pyruvate_**  **(Cmol Cmol^-1^)** |
| --- | --- | --- | --- | --- | --- |
| H16∆C-p2 | 0.057 ± 0.023 | 0.005 ± 0.002 | N.D. | 0.007 ± 0.000 | 0.339 ± 0.062 |
| H16∆C-p304 | 0.100 ± 0.041 | 0.013 ± 0.002 | 0.046 ± 0.002 | 0.089 ± 0.056 | N.D. |

**Supplementary Figures**

**Supplementary Figure 1.** Multiple sequence alignments using ClustalX (Thompson JD, Gibson TJ, Plewniak F, Jeanmougin F, Higgins DG. (1997). The CLUSTAL_X windows interface: flexible strategies for multiple sequence alignment aided by quality analysis tools. Nucleic Acids Res., 25, 4876-4882). Amino acids sequence corresponding from 215 to 287 residues of PduP from *Rhodopseudomonas palustris* (RpPduP_NADH) aligned to engineered NADH-dependent PduP variant and Bld from *C. saccharoperbutylacetonicum*.


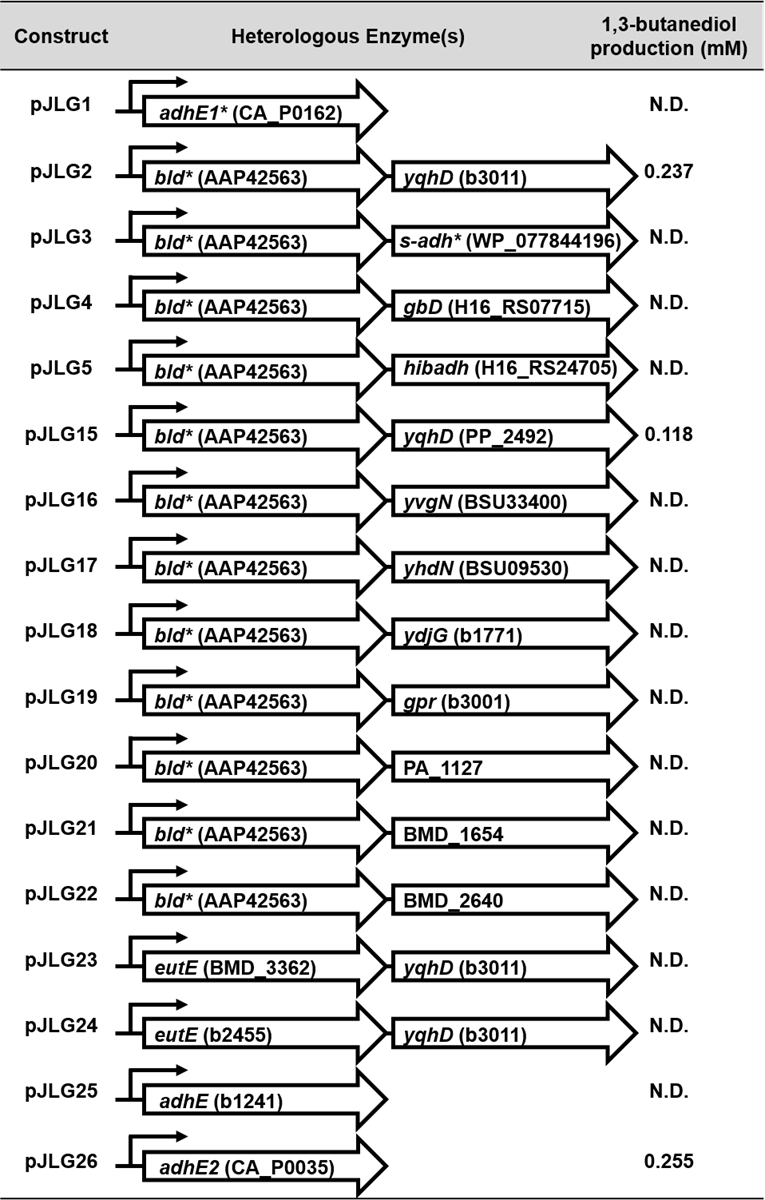


**Supplementary Figure 2.** *(R)*-1,3-BDO production in PHB negative *C. necator* H16 strain transformed with plasmid constructs containing genes that encode enzymes with butanal dehydrogenase and aldehyde reductase activities. Cells were cultivated in 10 mL 2 % (w/v) sodium gluconate nitrogen limiting minimal media. Samples were taken 70 h after induction of gene expression with 0.1 % (w/v) arabinose. * denotes codon optimised gene. N.D. denotes no 1,3-BDO detected by HPLC-RI analysis.


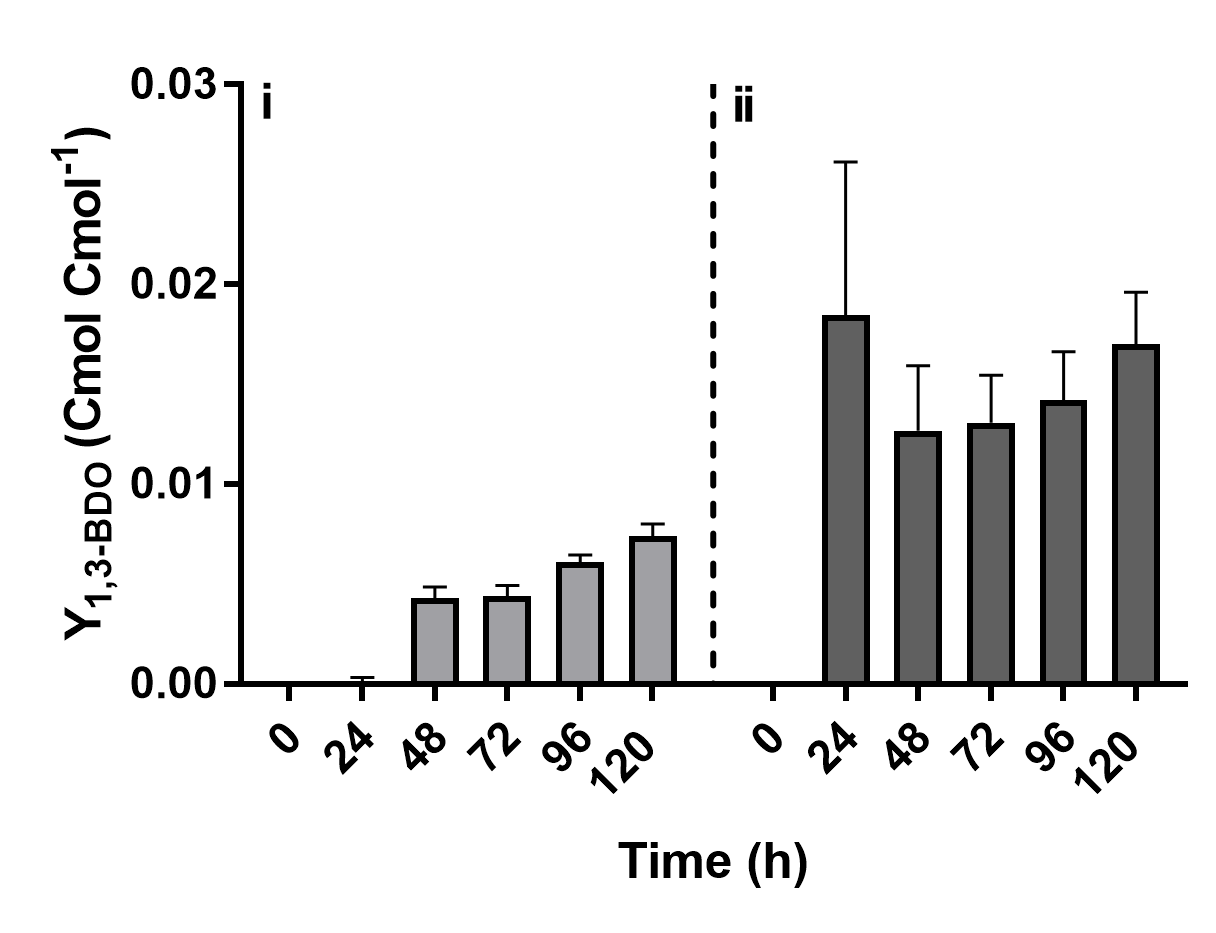


**Supplementary Figure 3.** Nitrogen limitation effect on (*R*)-1,3-BDO yield using *C. necator* strain H16∆C-p2. Cells were grown in 25 mL of either non-nitrogen-limiting MM with 2 % (w/v) sodium gluconate and 5 g/L NH_3_Cl (C:N = 6) or NLMM with 2 % (w/v) sodium gluconate and 0.6 g/L NH_3_Cl (C:N = 50), both supplemented with 0.01 % (w/v) L-arabinose and in 250 mL baffled shake flasks. Error bars represent standard deviation from three biological replicates.


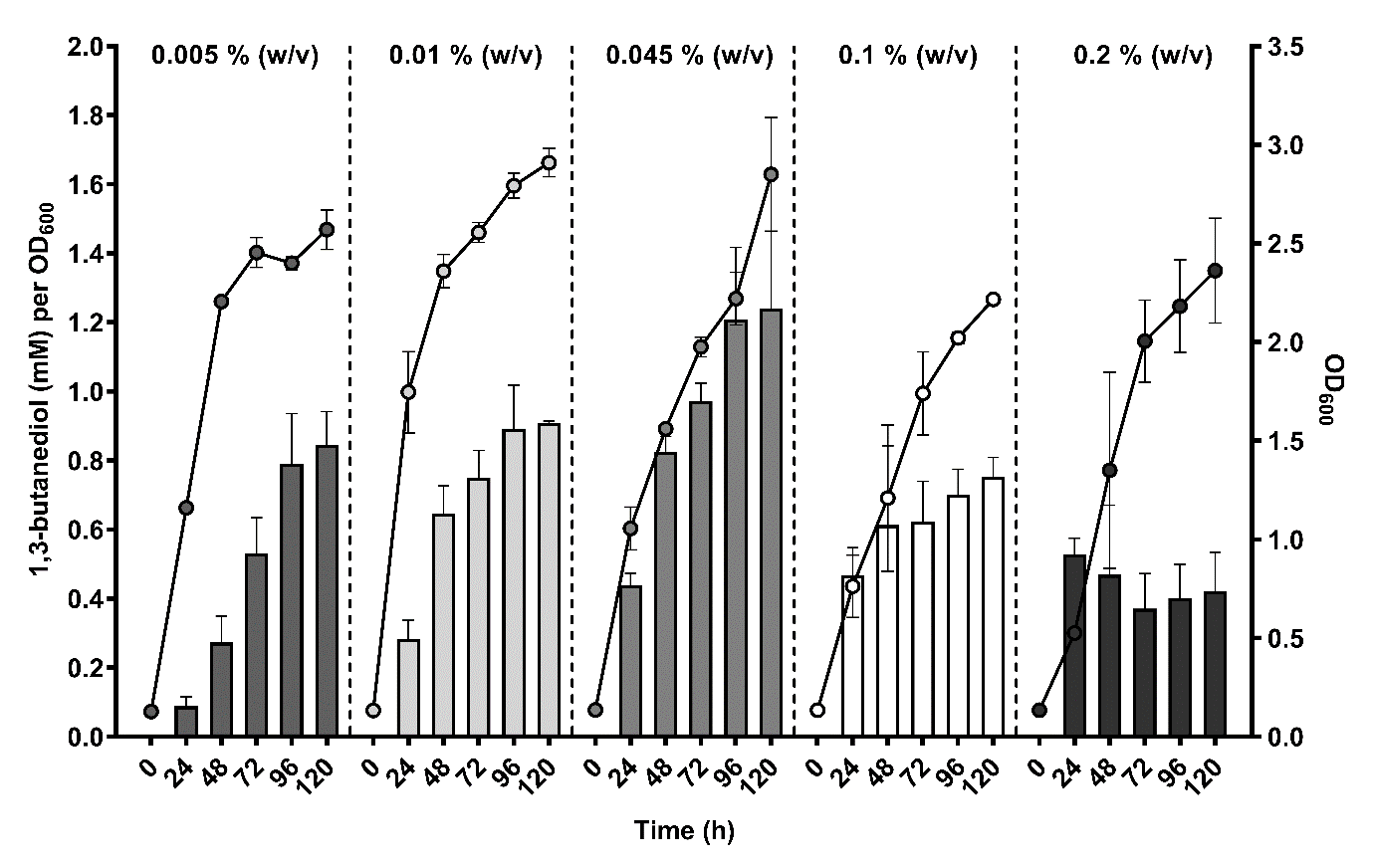


**Supplementary Figure 4.** L-Arabinose effect on growth (OD_600_, circles) of *C. necator* strain H16∆C-p2 and (*R*)-1,3-BDO titer (bars). Cells were grown in 25 mL of NLMM with 2 % (w/v) sodium gluconate in 250 mL baffled shake flasks. L-Arabinose was supplemented to final concentration of 0.005, 0.01, 0.045, 0.1 and 0.2%. Error bars represent standard deviation calculated from two biological replicates.


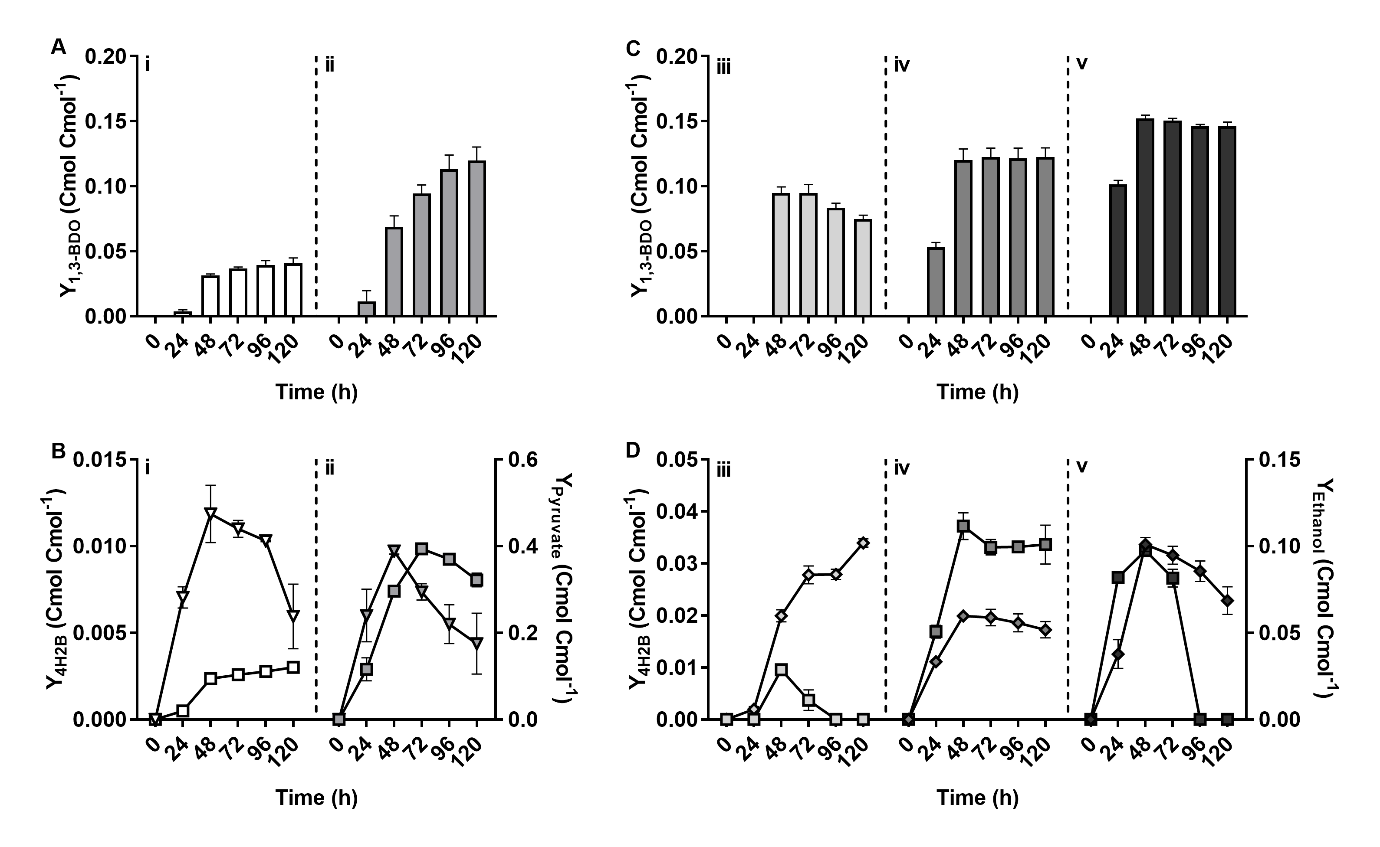


**Supplementary Figure 5.** Impact of gene copy number on the *(R)-*1,3-BDO production. *(R)-*1,3-BDO (solid bars) and by-product (4H2B (squares), ethanol (diamonds), and pyruvate (upside down triangles) yields determined for strains H16∆C_p2 (i) and H16∆1::54_p14 (ii) carrying 3HBCoA-dependent pathway (A and B) and strains H16∆C_p304 (iii), H16∆1::56_p14 (iv) and H16∆1::56_p45 (v) combining the 3HBCoA-dependent and pyruvate-dependent pathways (C and D). Cells were grown in NLMM supplemented with 2 % (w/v) sodium gluconate in 250 mL baffled shake-flasks. The biosynthetic pathway gene expression was induced by addition of 0.05 % (w/v) arabinose. Results represent the average of at least two biological replicates and error bars show standard deviation.

**Supplementary References**

Alagesan, S., Hanko, E. K. R., Malys, N., Ehsaan, M., Winzer, K., Minton, N. P., 2018. Functional genetic elements for controlling gene expression in *Cupriavidus necator* H16. Applied and Environmental Microbiology 84, e00878-18.

Bitinaite, J., Rubino, M., Varma, K. H., Schildkraut, I., Vaisvila, R., Vaiskunaite, R., 2007. USER™ friendly DNA engineering and cloning method by uracil excision. Nucleic Acids Research 35**,** 1992-2002.

Hanko, E. K. R., Minton, N. P. & Malys, N., 2017. Characterisation of a 3-hydroxypropionic acidinducible system from *Pseudomonas putida* for orthogonal gene expression control in *Escherichia coli* and *Cupriavidus necator*. Scientific Reports 7, 1724.

Lenz, O., Friedrich, B. 1998. A novel multicomponent regulatory system mediates H_2_ sensing in *Alcaligenes eutrophus*. Proceedings of the National Academy of Sciences of the United States of America 95 (21), 12474-12479.
